# Cognitive flexibility is associated with the age of onset and duration among patients with type 1 diabetes

**DOI:** 10.1038/s41598-025-99678-2

**Published:** 2025-07-07

**Authors:** Ding Mojun, Yuan Dongling, He Jing, Zou Wenjing, Li Xia, Li Chuting, Zhu Xiongzhao

**Affiliations:** 1 Medical Psychological Center, The Second Xiangya Hospital, Central South University, Changsha, 410011 Hunan China; 2 Medical Psychological Institute of Central South University, Central South University, Changsha, 410011 Hunan China; 3 National Clinical Research Center for Mental Disorders, Changsha, 410011 Hunan China; 4 National Center for Mental Disorder, Changsha, 410011 Hunan China; 5 Department of Psychology, Hunan First Normal University, Changsha, 410205 Hunan China; 6 Department of Radiology, The First Affiliated Hospital, Zhejiang University School of Medicine, Hangzhou, 310003 Zhejiang China; 7 National Clinical Research Center for Metabolic Diseases, Key Laboratory of Diabetes Immunology, Ministry of Education, and Department of Metabolism and Endocrinology, Second Xiangya Hospital, Central South University, Changsha, 410011 Hunan China

**Keywords:** Type 1 diabetes, Cognitive function, Age of diabetes onset, Duration, Glycemic fluctuation, Psychology, Type 1 diabetes

## Abstract

Cognitive impairment is a recognized risk in patients with type 1 diabetes, yet its contributing factors and overall impact remain insufficiently understood. This study aimed to explore the relationships between specific cognitive functions—namely memory, attention, and cognitive flexibility—and clinical characteristics, including age of onset and diabetes duration, as well as glycemic factors, such as glycemic control and extreme glycemic events, in a cohort of 331 patients with type 1 diabetes. Cognitive performance was assessed using the Wechsler Memory Scale, Wisconsin Card Sorting Test, and the Sustained Attention to Response Task. Network analysis revealed that age of onset and diabetes duration were central nodes in the network, strongly influencing cognitive flexibility. Additionally, age of onset was associated with a history of severe hypoglycemia, while diabetes duration was linked to visual memory decline. Significant differences were observed between the network structures of the adult-onset and childhood-onset groups, with the childhood-onset group showing greater interconnectedness. These findings emphasize the critical impact of age of onset and disease duration on cognitive outcomes in type 1 diabetes. Longitudinal studies are needed to further understand these relationships and guide targeted interventions to preserve cognitive function.

**Supplementary Information:**

The online version contains supplementary material available at 10.1038/s41598-025-99678-2.

## Introduction

Type 1 diabetes is a chronic metabolic disease characterized by the destruction of pancreatic beta cells, leading to an absolute lack of insulin. Patients need to rely on long-term insulin therapy to maintain blood sugar stability^1^. With advances in medical care and increased health awareness, the health-related quality of life of people with type 1 diabetes has significantly improved^2^. Meanwhile, the long-term complications have increasingly gained attention, with cognitive impairment being an important yet often overlooked complication of type 1 diabetes^3^.

Patients with type 1 diabetes may experience cognitive impairments, particularly in attention, memory, executive function, and processing speed.^4^. Among these, cognitive flexibility, an essential aspect of executive function involving the ability to adapt thinking and behavior in response to environmental changes, has attracted growing research interest^5^. Previous studies have reported that individuals with type 1 diabetes exhibit deficits in cognitive flexibility, as evidenced by increased perseverative errors and set-shifting difficulties in the Wisconsin Card Sorting Test compared to healthy controls^6^. All these cognitive impairments can, in turn, significantly affect patients’ daily functioning, disease management, and overall quality of life^7^. For instance, Palomo-Osuna et al. demonstrated that cognitive impairments could lead to reduced independence in daily life, increased risk of depression and anxiety, and negative impacts on social interactions and occupational performance^8^.

There is considerable evidence linking clinical characteristics of type 1 diabetes with cognitive impairment, with age of onset and disease duration being among the most frequently investigated factors. Type 1 diabetes can develop at any age, its peak incidence is during early adolescence^9^. Several studies have shown that early-onset patients (≤ 6 years old) exhibit more pronounced impairments in attention, executive function, and information processing speed, while juvenile patients tend to experience greater impairments in learning and memory. In contrast, adults with type 1 diabetes showed more significant declines in executive function and information processing speed^10^. Generally, adults with type 1 diabetes experienced relatively milder cognitive impairments. The disease duration is another critical factor, as it reflects the cumulative impact of metabolic disturbances, and its association with cognitive function is well-documented.

As metabolic control worsens, individuals with high HbA1c levels and severe hypoglycemia episodes might face significant cognitive challenges^11^. Moreover, Glycemic control behaviors also vary by age of onset and current age^10^. Fluctuating blood glucose levels, including hypoglycemia and hyperglycemia, are associated with both acute and chronic brain damage, adversely affecting cognitive function. Empirical studies indicated that repeated diabetic ketoacidosis episodes in children with type 1 diabetes were independently associated with lower IQ and memory deficits^12^. Additionally, severe hypoglycemic episode has been shown to increase the risk of cognitive dysfunction, particularly in memory, psychomotor speed, and mental efficiency^11^. Chronic hyperglycemia is similarly associated with cognitive impairments, including memory issues, learning difficulties, attention disorders, and neuropathy^13^. These findings underscored the importance of blood glucose control in maintaining cognitive health and suggested that better glycemic management could help slow cognitive decline, regardless of the age of onset.

However, much existing literature on cognitive function in type 1 diabetes patients has focused on specific glycemic parameters and extreme events like age of onset in adulthood versus childhood, with inconsistent findings^7,11^. One possible reason for these inconsistencies may lie in the lack of a comprehensive approach to exploring the complex relationships between clinical and cognitive factors.

Network analysis has emerged as a robust methodological framework for investigating complex, multivariate relationships in medical and cognitive research, offering a more integrative perspective than conventional statistical approaches^14^. Unlike traditional models, which primarily assess linear associations between individual variables, network analysis enables the simultaneous evaluation of multiple interrelated factors, providing a more integrative approach to understanding cognitive function in type 1 diabetes (T1D). This method allows for the identification of key variables most strongly associated with cognitive outcomes while capturing the broader structural patterns of interactions among cognitive functions, clinical characteristics, and glycemic parameters. By considering both direct and indirect associations, network analysis offers a systems-level perspective on these interrelations, rather than treating them in isolation. In this study, we employed network analysis to construct a model integrating multiple cognitive domains and diabetes-related factors, aiming to uncover novel associations that may have been overlooked in previous research. This approach provides a more nuanced understanding of how clinical and glycemic characteristics contribute to cognitive function in T1D.Taken together, this study aimed to comprehensively explore the complex relationships between cognitive functions—specifically memory, attention, and cognitive flexibility—and various clinical and glycemic characteristics in type 1 diabetes patients, including age of onset, disease duration, glycemic metabolism parameters, and extreme glycemic events, using network analysis. To our knowledge, this has not yet been investigated. The study had two primary objectives. The first was to examine the detailed structure and connectivity characteristics of the network that links cognitive functions with clinical and glycemic characteristics in type 1 diabetes. Through this analysis, key clinical variables related to type 1 diabetes that significantly impact patients’ cognitive functions were identified, as well as cognitive function indicators that are particularly sensitive to age of onset, disease duration, glycemic parameters and extreme glycemic events. The second objective involved constructing distinct networks for patients grouped by different ages of onset and life stages. These networks were analyzed to identify differences and unique characteristics specific to each group, providing a more comprehensive and dynamic understanding of the clinical features and treatment priorities for type 1 diabetes patients with different chronological disease indicators.

## Research design and methods

### Participants

This cross-sectional study was conducted between November 2015 and March 2022, with all data collected during a single outpatient visit. A total of 345 patients with type 1 diabetes mellitus were recruited from the Diabetes Clinic at the Second Xiangya Hospital in Changsha, Hunan, China, during regular visits. Fourteen patients declined participation. The final sample comprised 331 patients (160 males and 171 females), aged 6.5 to 52 years (M = 18.19, SD = 8.72). All assessments—including cognitive function evaluation, clinical history collection, and glycemic parameter measurement—were performed at a single time point. Although some patients had medical records from previous visits, only data from the designated study visit were included in this analysis to maintain the cross-sectional nature of the study. Diagnoses were confirmed by two endocrinologists using specific biomarkers, such as the levels of pancreatic autoantibodies, in accordance with the Chinese Type 1 Diabetes Guidelines and the American Diabetes Association’s criteria. Exclusion criteria included psychiatric or neurological disorders, hypertension, other chronic or acute diseases, premature birth (< 36 weeks gestation), birth complications, or physical limitations affecting testing. Additionally, participants with an IQ score below 70 were excluded to ensure their ability to complete the cognitive assessments reliably. This study was approved by the Ethics Committee of the Second Xiangya Hospital of Central South University. All procedures were performed in accordance with the ethical standards of the institutional research committee and with the 1964 Helsinki Declaration and its later amendments. All participants signed written informed consent forms prior to their participation in the study.

### Measures

#### Clinical and glycemic characteristics

Researchers collected demographic and clinical data using a designed form and medical records of participants’ initial outpatient consultations at The Second Xiangya Hospital. Variables included age, sex, years of education, age of type 1 diabetes onset, diabetes duration, blood glucose level, average hemoglobin A1c (HbA1c) levels, fasting blood glucose (FBG) levels, fasting C-peptide levels, history of severe hypoglycemia (SH), and history of diabetic ketoacidosis (DKA). The age of onset was defined as the time of diagnosis^15^. HbA1c levels, obtained from medical records, were averaged across all available test results prior to the study visit to account for interindividual variability in follow-up schedules^16^. Rather than being used as an independent variable in network analysis, mean HbA1c was incorporated into the calculation of the hyperglycemia exposure index (HEI), which reflects the cumulative glycemic burden over time. The HEI was calculated by converting each participant’s mean HbA1c and disease duration into Z-scores, which were then summed to generate a composite measure of hyperglycemic exposure:

$$HEI = {Z}_{HbA1c}+ {Z}_{disease duration}$$

where:

$${Z}_{HbA1c}=\frac{individual HbA1c-{M}_{HbA1c}}{{S}_{HbA1c}}$$

$${Z}_{disease duration}=\frac{individual disease duration-{M}_{disease duration}}{{S}_{disease duration}}$$

M and S refer to the mean and standard deviation of HbA1c and disease duration within the study population^17,18^.This approach accounts for interindividual differences in disease chronicity, ensuring that HEI captures the sustained impact of hyperglycemia beyond single-point HbA1c measurements. SH was defined as low blood glucose requiring assistance or glucagon injection due to seizure, loss of consciousness, or disorientation. DKA history was determined by medical records and by parents’ or guardian’ reports of hospitalizations and emergency department visits for DKA at other facilities. The biochemical criteria for the diagnosis of DKA include hyperglycemia with serum pH < 7.30. For youth with DKA, we recorded the serum pH and HbA1c at diagnosis. The medical record showed that all patients were corrected acidosis within 24 h. Blood glucose levels were measured before cognitive testing, with snacks or insulin administered as needed to maintain levels between 4 and 17 mmol/L^3,19^.

#### Cognitive functions

Cognitive function was assessed using the Wechsler Intelligence Scale, Wechsler Memory Scale (WMS), Wisconsin Card Sorting Test (WCST), and Sustained Attention to Response Task (SART). Specifically, general intellectual ability was evaluated with the Wechsler Intelligence Scale for Children—Chinese Revision for participants under 16 years of age, and the Wechsler Adult Intelligence Scale—Chinese Revision for those 16 years or older. These assessments are highly correlated (r > 0.9). Intelligence Quotient was calculated from five subtests: Information, Digit Span, Similarities, Picture Completion, and Block Design. The full-scale IQ score was computed by summing age-scaled subtest scores. The Similarities subtest evaluated abstract thinking and visual reasoning, while the Block Design subtest assessed spatial visualization and problem-solving abilities. The WMS assessed logical and visual memory, with participants asked to recall stories and reproduce drawings both immediately and after a delay. The WCST, which measured executive function, involved card sorting tasks, with key outcome measures being the number of categories completed and the number of perseverative errors, both reflecting cognitive flexibility. Finally, attention was evaluated using the SART, where participants responded to digits displayed on a screen. This test was administered only to participants in the Childhood-Onset vs. Adult-Onset subgroups. As a result, sustained attention measures were only available for this specific network analysis and were not included in other network comparisons. Further details of all these assessments are available in Table S1.

Table 1 summarizes all the instruments and measurement variables derived from each instrument that were utilized as network nodes in the later analyses.

**Table 1:** Overview of all the instruments and variables (nodes).

Domains and instruments	Nodes
Cognitive function	
Cognitive flexibility	
Modified Wisconsin Card Sorting Test, M-WSCT	Perseverative errors (WCST_P)
	Categories completed (WCST_C)
Memory	
Wechsler Memory Scale, WMS	Visual memory immediate test score (VM_1)
	Visual memory delay test score (VM_2)
	Logical memory immediate test score (LM_1)
	Logical memory delay test score (LM_2)
Sustained attention	
Sustained Attention to Response Task, SART	Intraindividual variability (VII)
	Omission error rate (O_R)
	Commission error rate (C_R)
	Mean reaction time (RT)
Visual reasoning and abstract thinking abilities	
Wechsler Intelligence Scale— Chinese Revision,WAIS-RC	The Similarity subtest of the WAIS-RC (IQ_S)
Spatial perception and processing abilities	
Wechsler Intelligence Scale— Chinese Revision,WAIS-RC	The Block Design subtest of the WAIS-RC (IQ_B)
Clinical and Glycemic Characteristics	
Clinical chronological indicators	Age of onset (A_O)
	Diabetes duration (D_D)
Glycemic parameters and extreme events	Diabetic ketoacidosis history (DKA)
	Severe hypoglycemia history (SHh)
	Fasting C-peptide levels (FCP)
	Hyperglycemic exposure index (HEI)*

Hyperglycemia exposure index score can reflect the impact of blood sugar control level and duration and is currently a widely used indicator to evaluate chronic hyperglycemia levels. In this study, all available HbA1c monitoring results and the patient’s disease duration were collected from the medical records of each child with type 1 diabetes to calculate each child’s hyperglycemic exposure index. The specific algorithm is: first, calculate the mean HbA1c of each patient based on all HbA1c values of each patient, and then convert the two variables of the patient’s mean HbA1c and disease duration into standard scores (z-score); Second, the hyperglycemic exposure index for each patient was obtained by adding the patient’s z-score over the course of the disease and the mean z-score for HbA1c.

### Statistical analysis

The data were preprocessed and analyzed using SPSS 26.0 for descriptive statistics. Descriptive statistics consist of means and SDs for continuous variables and numbers and frequencies for categorical variables. Network analyses and comparisons were conducted with R, version 4.0.5. The network analysis approach followed the guidelines by Epskamp et al.^20^**.** A significance level of 0.05 was used.

Missing values were analyzed and estimated using the Expectation–Maximization Algorithm (EM) in SPSS, a widely used approach for maximum likelihood estimation in the presence of missing data. Assumption was made that the data were missing at random (MAR), which is a reasonable assumption for the context of present study. The imputation process consisted of two main steps: the expectation (E) step, where the missing values were predicted based on the observed data, and the maximization (M) step, where model parameters were updated to reflect the newly imputed values. The algorithm was run for 50 iterations, with convergence assessed based on the changes in parameter estimates between consecutive iterations. In total, imputation was performed on 9 continuous variables that had varying degrees of missingness. Prior to imputation, descriptive statistics were calculated to understand the patterns of missingness. After imputation, we verified the quality of the imputations by examining the distributions and comparing them with the observed values^21^. Further details concerning missing values are shown in supplementary materials Figure S1.

Network analysis was primarily used as a descriptive tool to explore the relationships among cognitive functions and clinical-glycemic characteristics. The analysis was performed on data from all 331 participants. Adult patients were divided into childhood-onset and adult-onset groups based on age of onset, with separate networks constructed for comparison. Additionally, networks for minor patients were compared with those of childhood-onset adult patients to explore developmental differences.

To control potential confounders, multivariable regression models were used before conducting the network analysis. These models adjusted for age, sex, disease duration, and other clinical variables (e.g., HbA1c levels, insulin usage) to ensure that the observed relationships in the network were not biased by confounding factors.

The standardized Gaussian graphical model network was estimated using the R-packages *bootnet* and *qgraph*. Nodewise errors, representing variance explained by connected nodes, were visualized using the *mgm* package. The graphical *LASSO* model with *EBICglasso* reduced the probability of false connections. Partial correlation networks corrected correlations between variables for all other variables, visualized as nodes and edges.

The Fruchterman Reingold algorithm detected node pairs with stronger interconnections and higher centrality indices. Centrality indices, including strength, expected influence (EI), closeness, and betweenness, were employed to assess the relative importance of nodes within the network^22^. Strength quantifies the direct number and weight of connections, while expected influence (EI) extends this by also considering the influence of a node’s connections beyond its immediate neighbors^23^. Given its stability and interpretability in psychological networks, Strength was selected as the primary index for node comparisons in this study^22^.Edge-weight accuracy was computed using *bootnet* with 1000 nonparametric bootstrap samples. The stability of network centrality measures was assessed using the correlation stability coefficient (CS-coefficient), which quantifies how reliably edge weights and node strength rankings remain consistent when a proportion of the data is removed. A CS-coefficient greater than 0.7 is considered highly stable, suggesting that network estimates remain robust even when up to 70% of the sample is removed. A CS-coefficient between 0.50 and 0.70 indicates moderate stability, whereas values below 0.50 suggest that network estimates may be more susceptible to sample variability^20^. Finally, bootstrapped difference tests were conducted to identify statistically significant differences between edge weights and node strengths^20,22^.

In the network constructed among 331 patients with type 1 diabetes, the role of individual items as bridges between communities was assessed. Nodes that connect different predefined domains are called bridge nodes, which act like a bridge to link the two communities in the network. Bridge strength, defined as the absolute sum of the edge weights connecting an item in one community to items in another community, and bridge expected influence, defined as the nonabsolute sum of all edges connecting an item to others in a different community, were calculated for each item. These metrics were computed separately for each cognitive function in relation to clinical and glycemic characteristics.

To assess differences between groups, comparisons were conducted using the *Network Comparison Test* (NCT) package in R, with 1000 permutation test. Global network strength, defined as the weighted sum of absolute connection values, reflects node interconnectivity. Significant differences (*p* < 0.05) between groups were identified, with specific edge-weight differences examined using Bonferroni correction for multiple comparisons.

## Results

### Descriptive statistics and item inspection

Detailed descriptive statistics for the entire sample and subgroups were provided in Table 2. Additionally, zero-order correlation matrices (Table S2-S7) illustrated the direct relationships between clinical and cognitive variables.

**Table 2:** Sample characteristics.

	Total type 1 diabetes patients (n = 331)	Adult patient (n = 150)		Child patient group (n = 181)
		Childhood-onset group (n = 78)	Adult-onset group (n = 72)	
Age, years (mean [SD])	18.19(8.72)	21.61(4.61)	28.87(7.41)	12.06(3.32)
Female sex (n, %)	171(51.6)	44(56.4)	34(43.6)	89(49.2)
Education, years (mean [SD])	9.89(4.97)	13.83(1.86)	14.83(2.56)	6.20(3.28)
Diabetes-related variables (mean [SD])				
Age of onset, years	14.54(8.16)	13.60(7.88)	26.92(6.77)	9.98(3.80)
Diabetes duration, years	3.66(4.45)	7.88(6.00)	2.91(3.55)	2.15(2.55)
DKA history, n	0.57(0.62)	0.56(0.50)	0.42(0.50)	0.63(0.70)
SH history, n	0.49(0.84)	0.37(0.49)	0.20(0.40)	0.66(1.03)
Mean HbA1c(%)	8.33(2.03)	8.39(2.10)	8.02(2.51)	8.42(1.87)
Fasting C-peptide levels, pmol/L		− 0.77(1.07)	-0.53(1.20)	
Hyperglycemic exposure index	0(1.32)	0.49(1.33)	0.09(0.92)	0(1.24)
Cognitive functions (mean [SD])				
Wechsler Memory Scale				
Visual memory immediate test score	9.95(2.81)	10.97(2.57)	10.25(2.73)	9.41(3.06)
Visual memory immediate test score	9.17(2.95)	10.51(2.72)	9.04(2.31)	8.53(3.11)
Logical memory immediate test score	6.91(2.08)	7.76(2.29)	6.87(2.98)	6.59(2.87)
Logical memory delay test score	5.89(2.71)	6.65(2.56)	5.47(2.73)	5.77(2.78)
Modified Wisconsin Card Sorting Test				
Perseverative errors	48.11(13.36)	47.57(8.36)	45.31(8.57)	50.02(15.44)
Categories completed	2.92(1.26)	3.10(1.29)	3.51(1.26)	2.60(1.18)
Sustained Attention to Response Task				
Omission error rate		3.03(4.98)	0.06(0.27)	
Commission error rate		8.37(7.44)	0.78(2.61)	
Mean reaction time		377.28(95.39)	388.79(89.32)	
Intraindividual variability		0.29(0.11)	0.25(0.09)	
Chinese version of the Wechsler adult intelligence, WAIS-RC				
Intelligence quotient, IQ		111.68(13.63)	113.53(13.06)	109.14(13.48)
The Similarity subtest of the WAIS-RC		13.03(3.76)		13.02(3.22)
The Block Design subtest of the WAIS-RC		16.03(10.45)		12.08(4.06)

DKA = diabetic ketoacidosis, SH = severe hypoglycemia, HbA1c = hemoglobin A1c. Data are mean (standard deviation) or frequency (%).

Table 2 presents the demographic, clinical, and cognitive characteristics of the study sample. The mean age of participants was 18.19 years (SD = 8.72), and 51.6% were female. Participants had an average education duration of 9.89 years (SD = 4.97). Regarding diabetes-related variables, the mean age of onset was 14.54 years (SD = 8.16), with a mean disease duration of 3.66 years (SD = 4.45). The history of diabetic ketoacidosis (DKA) and severe hypoglycemia (SH) was recorded for all participants. The mean HbA1c level for the total sample was 8.33% (SD = 2.03), and fasting C-peptide levels were generally low. In the cognitive assessments, Wechsler Memory Scale (WMS) results showed that visual memory immediate test scores had a mean of 9.95 (SD = 2.81) across all participants. Logical memory performance was assessed through immediate recall (M = 6.91, SD = 2.08) and delayed recall (M = 5.89, SD = 2.71). For executive function, measured by the Wisconsin Card Sorting Test (WCST), participants exhibited an average of 48.11 (SD = 13.36) perseverative errors and completed an average of 2.92 (SD = 1.26) categories. Sustained attention, as measured by SART, showed an omission error rate of 3.03 (SD = 4.98) in childhood-onset adult patients and 0.06 (SD = 0.27) in adult-onset patients. Intelligence was assessed using WISC-RC (for participants aged 6–16 years) or WAIS-RC (for participants aged 17–18 years). The Similarities subtest scores were 13.03 (SD = 3.76) for childhood-onset adult patients and 13.02 (SD = 3.22) for child patients. Block Design scores were 16.03 (SD = 10.45) for childhood-onset adult patients and 12.08 (SD = 4.06) for child patients.

These descriptive statistics provide an overview of the cognitive performance and clinical characteristics within the study population. Given the study’s focus on network analysis, individual test score distributions are presented without statistical comparisons.

### Cognitive functions, clinical and glycemic characteristics network

**Fig. 1:**
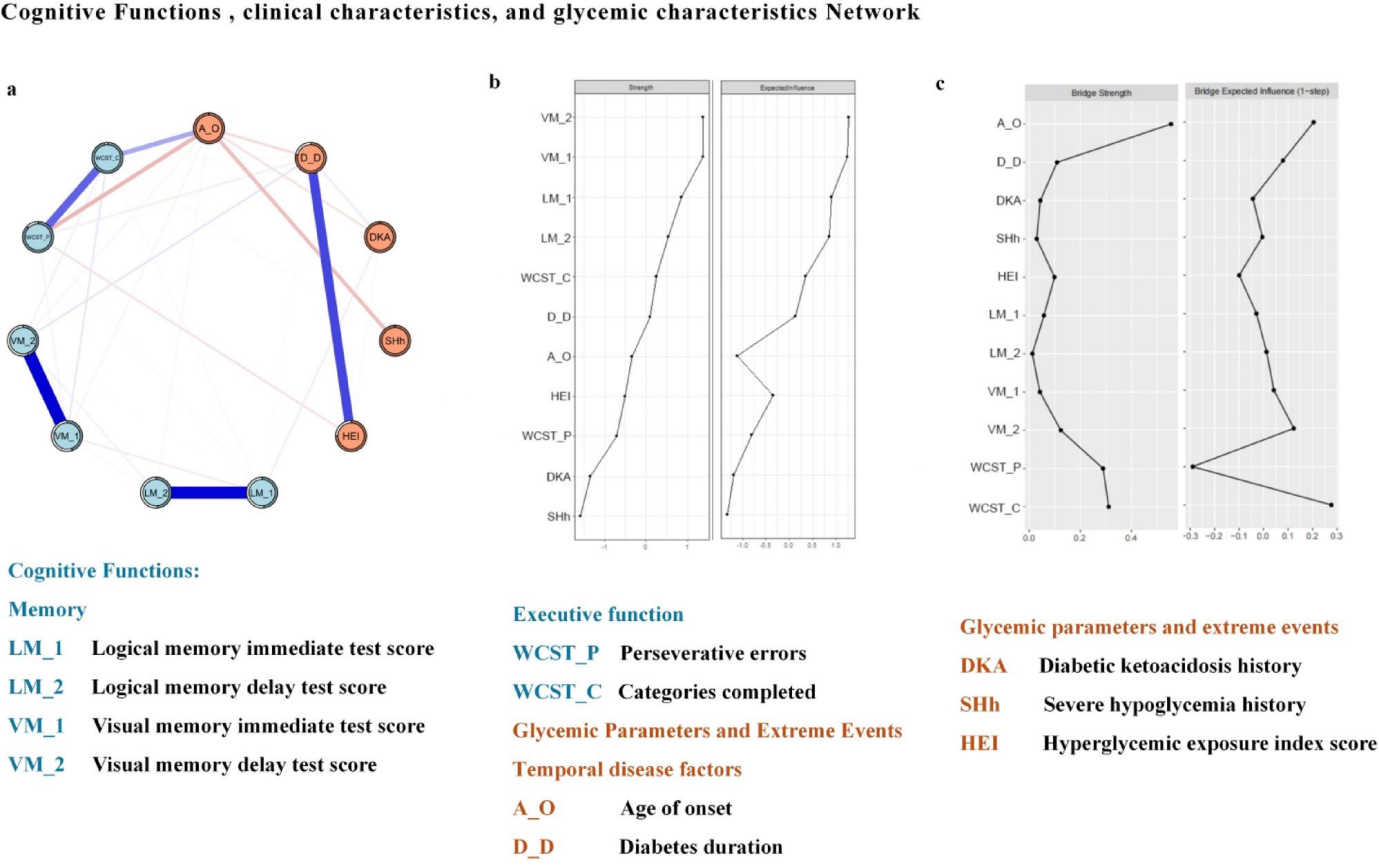
The estimated network with cognitive functions and clinical-glycemic characteristics is shown (**a**). Each circular node represents a clinical-glycemic characteristics or a score of cognitive testing. The edge (line) connecting nodes represents partial polychoric correlations, with thicker, more saturated edges denoting stronger connections, blue edges denoting positive relationships, and red edges denoting negative relationships. The centrality indices, including strength and expected influence (EI), are shown as standardized z scores. (**b**). Nodes with high strength play a critical role in shaping the relationships among other nodes, while nodes with high expected influence have more positive connections to other nodes. The bridge strength and bridge expected influence of each node is shown as a standardized z score (**c**).

The network included 331 patients, as illustrated in Fig. 1a. It contained 51.2% nonzero edges, with edge weights ranging from − 0.229 to 0.827. The edge weights matrix and an overview of the number of edges are in Table S8. Centrality indices (strength & expected influence (EI)) are presented as standardized z scores in Fig. 1b. Nodes with greater strength were more interconnected, while those with higher expected influence had a greater impact beyond their immediate connections.

### Edge weight and centrality accuracy

Figure 1b shows the centrality indices of the network. The age of onset, diabetes duration, and visual memory delay test score had the highest centrality (near 1), with 8, 7, and 7 connected edges, respectively. This indicates that the age of onset was pivotal in the network. There were 24 edges with weights of 0.280, 0.229, and 0.204, respectively. The CS-coefficients of the edge and strength were 0.749, indicating high stability, meaning that the relative order of edge weights and node strengths remains reliable even when up to 75% of the data is removed. However, while this coefficient confirms statistical robustness, it does not account for unmeasured confounders or data dependencies. Therefore, small differences in edge weights should still be interpreted with caution, especially for edges with near-zero weights, where bootstrapping variability may introduce uncertainty. Similarly, node strength rankings may also be subject to variability, particularly for nodes with lower centrality values, where small changes in network structure could affect their relative positioning.

### Bridges

The bridge item between cognitive functions and clinical and glycemic characteristics was identified through bridge centrality. Three nodes (A_O, WCST_C and WCST_P) were identified with top-ranked bridge strengths. The bridge strength of each item is shown in Fig. 1c. The CS-coefficient for bridge strength was 0.517, suggesting moderate stability. This implies that while bridge node rankings remain relatively consistent, they should be interpreted with some caution. The edge connecting “WCST_P” had the highest edge weight (0.507) with node “WCST_C” in the cognitive functions community, while the edge connecting “A_O” had the highest edge weight (0.280) in the clinical and glycemic characteristics community.

### Network comparisons

#### Effect of age of onset: childhood-onset versus adult-onset

**Fig. 2:**
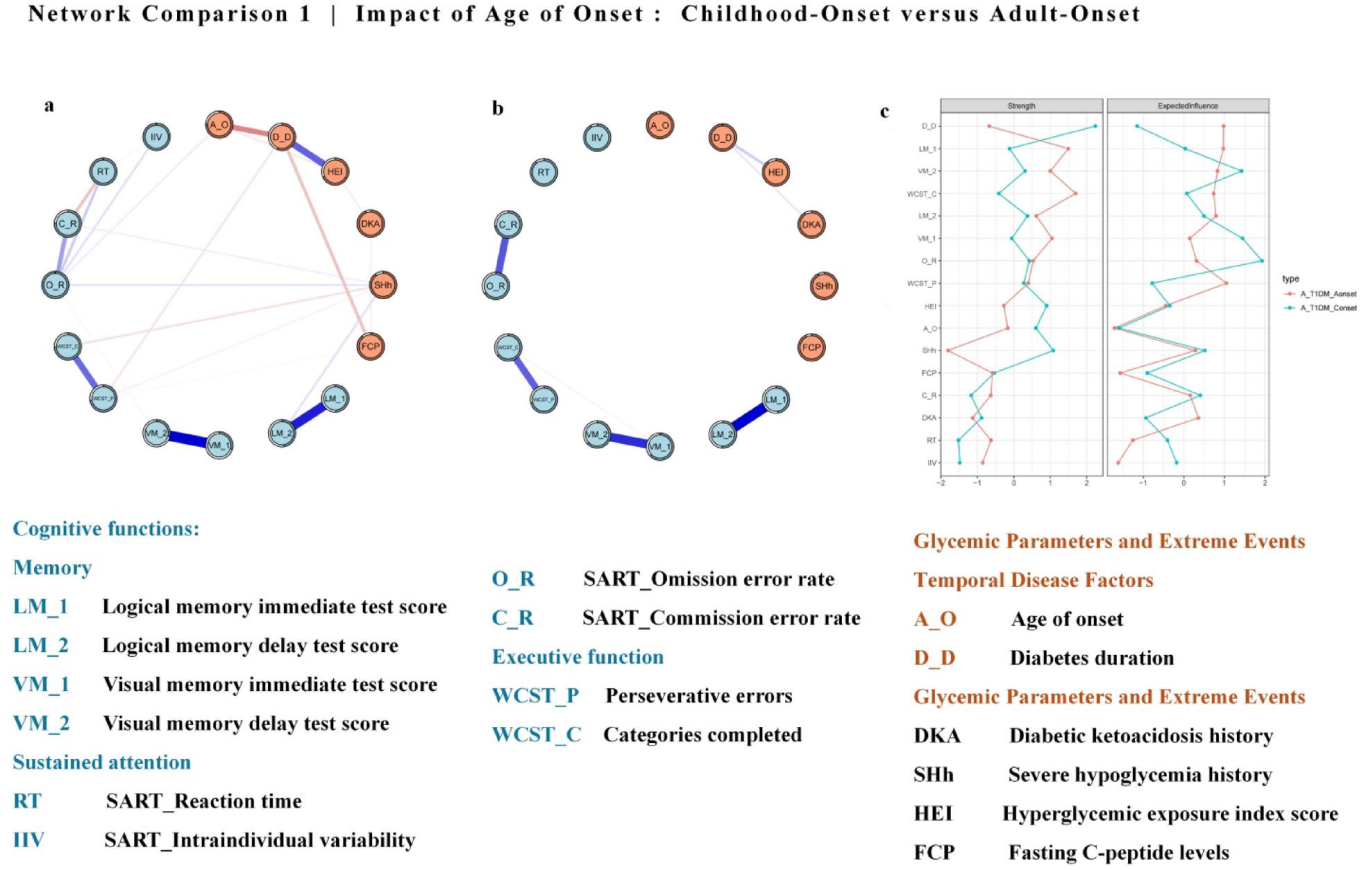
Estimated networks with cognitive functions and diabetes-related variables are shown. (**a**: Childhood-onset group; **b**: Adult-onset group). The centrality indices, including strength and expected influence (EI), are shown as standardized z scores. (c: Blue-Childhood-onset group; Red-Adult-onset group). Each circular node represents a diabetes-related variable or a score of cognitive testing. The edge (line) connecting nodes represents partial polychoric correlations, with thicker, more saturated edges denoting stronger connections, blue edges denoting positive relationships, and red edges denoting negative relationships. Nodes with high strength play a critical role in shaping the relationships among other nodes, while nodes with highexpected influence have more positive connections to other nodes.

As illustrated in Fig. 2a,b, separate networks for childhood-onset (n = 78) and adult-onset adult (n = 72) patients to identify differences. Detailed networks and edge weights are shown in the Supplementary Materials (Figure S5-S7, Table S9-S10). Figure 2c shows the centrality indices of the networks. In the childhood-onset group, diabetes duration and severe hypoglycemia history emerged as significant nodes, with diabetes duration linked to FCP levels and severe hypoglycemia linked to logical memory delay and WCST categories. Stability was confirmed with a CS-coefficient of 0.44 for both edge weights and strength in the childhood-onset group, and 0.36 for edge weights and 0.28 for strenghth in the adult-onset group. A CS-coefficient below 0.50 suggests that network centrality rankings may be less stable, particularly in the adult-onset group, where lower values indicate greater potential variability in network estimates.

The Network Comparison Test revealed significant differences in network structure (M = 0.37, *p* = 0.02) and overall connectivity (S = 2.59, *p* = 0.00), with higher connectivity in the childhood-onset group (4.45 vs. 1.86, *p* < 0.05).

#### Effect of current age in childhood-onset type 1 diabetes: child versus adult

**Fig. 3:**
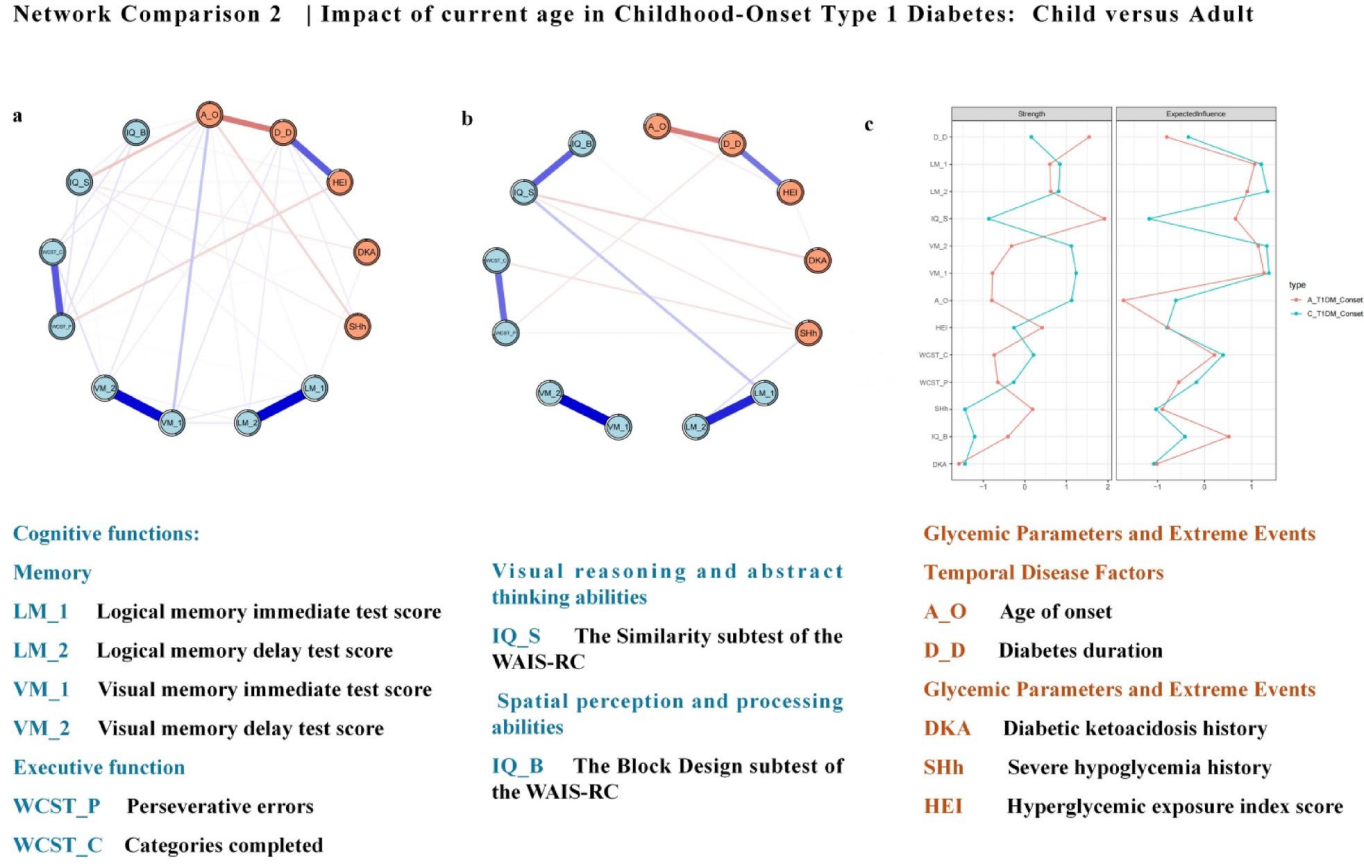
Estimated networks with cognitive functions and diabetes-related variables are shown. (**a**: Child group; **b**: Adult group). The centrality indices, including strength and expected influence (EI), are shown as standardized z scores. (**c**: Blue-Child group; Red-Adult group). Each circular node represents a diabetes-related variable or a score of cognitive testing. The edge (line) connecting nodes represents partial polychoric correlations, with thicker, more saturated edges denoting stronger connections, blue edges denoting positive relationships, and red edges denoting negative relationships. Nodes with high strength play a critical role in shaping the relationships among other nodes, while nodes with high expected influence have more positive connections to other nodes.

A network comparison was also conducted between child (n = 181) and adult (n = 78) groups among childhood-onset patients (Fig. 3a,b). Detailed networks and edge weights are shown in the Supplementary Materials (Figure S8-S10, Table S13-S16). Figure 3c shows the centrality indices of the networks. The child group’s network highlighted the age of onset and visual memory test scores as important nodes. The age of onset was linked to visual memory immediate test scores and the Wechsler Intelligence Scale similarity subtest. The hyperglycemia exposure index was linked to WCST perseverative errors. In the adult group, significant nodes were the similarity subtest score and diabetes duration, with the similarity subtest score linked to logical memory immediate test scores and DKA history. Stability was confirmed with a CS-coefficient of edge at 0.75 and strength at 0.67 for the child group. In contrast, the adult group showed lower stability, with a CS-coefficient of 0.51 for edge weight and 0.44 for node strength. These results suggest that the childhood-onset adult group’s network structure may be less stable than the child group’s network, requiring cautious interpretation of specific centrality measures.

The Network Comparison Test revealed significant differences in network structure (M = 0.42, *p* = 0.02) but no significant differences in global strength (S = 1.14, *p* = 0.63). This suggests substantial structural differences between child and adult groups, though global strength showed some similarities.

## Discussion

The present study is the first to apply network analysis to examine the intricate relationships among cognitive functions, clinical characteristics, and glycemic factors in patients with type 1 diabetes (T1D). Unlike previous studies that have primarily focused on isolated glycemic markers—such as HbA1c levels, severe hypoglycemia, or disease duration—this study employs a network-based approach to simultaneously assess the interactions between multiple factors in shaping cognitive function. By integrating these variables within a single analytical framework, network analysis provides a more comprehensive perspective on the multivariate relationships that contribute to cognitive outcomes in T1D.

Through an analysis of 331 participants, the study identified age of onset and diabetes duration as the most central and bridge nodes within the network of cognitive functions, clinical characteristics, and glycemic factors in patients with type 1 diabetes. These two clinical variables were strongly associated with cognitive flexibility, as indicated by their robust connections to WCST Perseverative Errors and Categories Completed. Importantly, cognitive flexibility measures also exhibited the highest bridge centrality within the cognitive domain, suggesting that cognitive flexibility plays a crucial role in integrating clinical and metabolic factors with cognitive outcomes in T1D. Moreover, significant associations were observed between age of onset and severe hypoglycemia history, as well as between diabetes duration and visual memory performance, reinforcing the role of these variables in shaping cognitive outcomes.

Comparative network analyses further underscored the differential effects of childhood-onset versus adult-onset type 1 diabetes, revealing distinct structural variations within the network of cognitive functions, clinical characteristics, and glycemic factors. These findings provide deeper insights into how early metabolic disturbances and prolonged glycemic fluctuations contribute to cognitive alterations across the lifespan. By leveraging network analysis, this study moves beyond traditional linear statistical approaches and highlights the importance of examining multidimensional interactions in understanding cognitive impairment in type 1 diabetes. These findings emphasize the need for future research to explore longitudinal changes in these networks and to develop targeted interventions aimed at mitigating cognitive decline in high-risk subgroups.

### Cognitive functions and clinical-glycemic characteristics network

The present study revealed that the age of onset plays a critical role in shaping the relationship between cognitive functions and clinical-glycemic characteristics in patients with type 1 diabetes. Childhood-onset diabetes was closely associated with more pronounced cognitive impairments, particularly in areas of memory, learning, and executive function. These impairments have been attributed to disruptions in insulin signaling and brain development, particularly in the hippocampus and frontal lobes, which were highly sensitive to metabolic disturbances^11^. In the current network analysis, a younger age of onset was identified to be associated with a longer disease duration, increased frequency of diabetic ketoacidosis (DKA) episodes, and greater glycemic exposure, which are all risk factors for prolonged hyperglycemia^12^. Chronic hyperglycemia is known to lead to the accumulation of glycation end products, oxidative stress, and microvascular damage, all of which contribute to brain insulin resistance and neurodegeneration^24^. These pathological changes have been associated with progressive reductions in gray and white matter volumes in critical brain regions responsible for cognitive functions^13,25^. Previous MRI studies have demonstrated long-term reductions in brain volume, particularly in the frontal and temporal lobes—regions essential for executive function and memory^10,26,27^. Consistent with these structural changes, current research has observed that a history of DKA is significantly correlated with poorer performance on logical memory tests. Furthermore, the hyperglycemia exposure index is closely linked to worse performance on the Wisconsin Card Sorting Test (WCST), which assesses cognitive flexibility. The present study also found a significant negative correlation between disease duration and perseverative errors, with longer disease duration being associated with fewer errors. This suggests that, over time, patients may develop better strategies or cognitive resilience to address executive function tasks. This adaptation may reflect a form of learning in which, despite the adverse effects of prolonged hyperglycemia, patients developed strategies to more effectively cope with task demands over time^28,29^.

In the present study, results of network analysis highlighted the age of onset as a central node directly influencing cognitive flexibility, demonstrated by its associations with the WCST Perseverative Errors and Categories Completed. In the network including all T1D patients (without categorizing based on age of onset or current age), an earlier age of onset was negatively correlated with perseverative errors and positively correlated with the number of categories completed. These findings suggest that patients diagnosed at a younger age may exhibit poorer cognitive flexibility, as indicated by more perseverative errors and fewer categories completed. Additionally, both the age of onset and cognitive flexibility served as key bridge nodes in the network, linking cognitive functions and clinical-glycemic characteristics. These findings underscored the cumulative impact of childhood-onset diabetes on cognitive flexibility, as well as the broader cognitive decline linked to prolonged disease duration. Our findings suggest that cognitive flexibility serves as a key mediator in the relationship between clinical factors and cognitive function in T1D. Given that cognitive flexibility was a central bridge node, it was possible that its impairment could have partially accounted for the broader executive function deficits observed in patients with an earlier age of onset. This aligned with previous studies suggesting that cognitive flexibility has played a fundamental role in adaptive reasoning and decision-making, both of which are crucial for effective diabetes self-management^30^.Previous neuroimaging studies have reported structural changes in the striatum and thalamus, alongside impairments in the mesial temporal cortex. However, no significant changes in cerebral blood flow alterations have been observed^31^. These changes in these brain regions may lead to deficits in executive function, particularly in tasks requiring memory and cognitive flexibility^32^. Furthermore, severe hypoglycemia is known to be a key factor influencing processing speed and executive function, especially in childhood-onset patients more vulnerable to glucose fluctuations. Importantly, these impairments in cognitive flexibility were highly relevant for diabetes management, as patients with higher cognitive flexibility tend to be more capable of problem-solving, adhering to treatment regimens, and maintaining stable glucose levels^33,34^. Given the critical role of cognitive function in diabetes self-management, greater clinical attention should be paid to identifying and addressing cognitive deficits in these patients, with targeted interventions aimed at enhancing cognitive flexibility and executive functioning to improve long-term metabolic outcomes.

### Effect of age of onset: childhood-onset versus adult-onset

Although the correlations between cognitive function and clinical-glycemic characteristics were evident across the network, the specific relationships varied by age of onset. Therefore, we further explored these factors in the following analyses.

A comparison between childhood-onset and adult-onset type 1 diabetes patients revealed significant differences in network structure, global strength, and node connections, which may have implications for tailoring treatment strategies based on age of onset. In childhood-onset patients, the network showed stronger connections between nodes representing disease duration, chronic hyperglycemia, and cognitive functions, suggesting that these factors may contribute to more pronounced cognitive impairments. Notably, the “SHh” node demonstrated connections with several cognitive function nodes in the childhood-onset group, a pattern that was absent in the adult-onset network. This increased connectivity of the “SHh” node suggested a stronger link between cognitive functions and diabetes-related factors in childhood-onset patients. As highlighted in previous research, such connections may contribute to more pronounced impairments in executive function, attention, visual-spatial abilities, and processing speed^35^. Early onset, particularly before the age of five, is linked to delayed motor skill development and reduced academic performance^36^. In contrast, adult-onset patients exhibited milder cognitive impairments, likely due to more stable brain energy metabolism and blood flow regulation during glucose fluctuations, as key stages of brain development were already complete^37^.

Severe hypoglycemia, a central node in the childhood-onset network, significantly impacted cognitive functions such as verbal memory and executive function. Frequent hypoglycemic episodes in younger patients have been associated with disruptions in brain metabolism, increased free radicals, and neuronal damage, particularly in the hippocampus and frontal lobes^13^. Consequently, recurrent episodes of hypoglycemia may contribute to chronic inflammation and further cognitive decline over time. Previous MRI studies have demonstrated that these brain regions essential for memory and reasoning are particularly vulnerable to damage caused by severe hypoglycemia^12,25,38^.

Additionally, metabolic differences between childhood-onset and adult-onset patients may help explain variations in cognitive outcomes. Childhood-onset patients exhibited a negative correlation between disease duration and fasting C-peptide levels. Childhood-onset patients typically present with lower C-peptide levels at diagnosis, suggesting poorer initial metabolic control compared to adult-onset patients, who typically exhibit higher C-peptide levels and more stable glucose regulation^7^. These metabolic differences, combined with more frequent glucose fluctuations in childhood-onset patients, contributed to greater brain structural changes and cognitive impairments^37^. Effective blood glucose management, particularly in minimizing extreme glucose events, is essential for preserving cognitive function in both childhood-onset and adult-onset patients, with early intervention being particularly important for those diagnosed in childhood^19^.

### Effect of disease duration: current age (child versus adult) in childhood-onset type 1 diabetes

A comparison between childhood-onset patients who were still children and those who had reached adulthood revealed distinct differences in network structure and connectivity patterns, even though overall network connectivity and edge strength remained relatively stable. In childhood-onset patients, cognitive function continued to be affected by blood glucose fluctuations into adulthood, with significant changes emerging over time. As a result, treatment and monitoring strategies should be adapted according to age. The age of onset served as a central node in childhood-onset patients, being strongly linked to performance on immediate visual memory and similarity tests. In contrast, for adult-onset patients, disease duration played a more prominent role, with diabetic ketoacidosis showing a stronger correlation with the similarity test. Long-term studies have suggested that childhood-onset patients experience persistent structural brain damage, which continues to affect cognitive function into adulthood^39^. Functional MRI studies in children with type 1 diabetes have consistently shown lasting damage in critical brain regions, including the prefrontal cortex and hippocampus, which are particularly susceptible during childhood development^40^. Although adults with childhood-onset diabetes may develop adaptive mechanisms to compensate for blood glucose fluctuations, poor metabolic control remains a significant risk factor for cognitive impairments^7^.

The present study also revealed a significant impact of repeated DKA episodes on the brain, particularly in regions crucial for memory, learning, and executive function, including the hippocampus, frontal lobes, and parietal lobes. MRI studies have shown that DKA led to reduced gray matter volume and white matter integrity in these regions, with childhood-onset patients experiencing more pronounced damage due to increased vulnerability during brain development^12^. This damage contributes to deficits in cognitive functions such as memory and attention^6,41^. Furthermore, mental health challenges, including anxiety and depression are more prevalent among childhood-onset patients, underscoring the need for comprehensive care and lifelong psychological support^41^.

### Strengths and limitations

This study is the first to apply network analysis to explore the relationships between cognitive functions and clinical-glycemic characteristics in type 1 diabetes, providing a broader perspective on how these factors interact. Unlike traditional regression models, which primarily assess linear associations, network analysis enabled the identification of key central and bridge nodes, revealing how age of onset and disease duration are positioned within the broader cognitive-glycemic network. By modeling these interconnections, our study offers a more integrated understanding of how clinical and metabolic factors relate to cognitive function in T1D. These findings also suggest that cognitive flexibility, as measured by WCST performance, plays a particularly relevant role in these associations. Given its position as a bridge node, cognitive flexibility appears to be a key variable linking clinical characteristics with executive function outcomes. While this study does not establish causality, these results highlight the potential importance of considering cognitive flexibility when examining the neurocognitive effects of T1D, particularly in relation to long-term disease management. Furthermore, by comparing childhood-onset and adult-onset T1D networks, our study provides insights into how age-related differences may shape cognitive outcomes. The stronger associations observed in childhood-onset patients suggest that early metabolic dysregulation could have lasting effects on cognitive flexibility and broader executive function. These findings underscore the need for future research to further explore developmental and disease-related factors contributing to cognitive variability in T1D.

Despite its strengths, the study has several limitations. First, the sample size was relatively small, especially after group division, and differences in group sizes might have limited the verification of some statistical associations. Additionally, all participants were Chinese, which limits the generalizability of the findings to other populations. Second, since the SART was only administered to participants in childhood-onset and adult-onset patient groups, its role in type 1 diabetes-related cognitive function could not be examined across all networks. This limitation restricts broader conclusions regarding the influence of sustained attention in this population. Future studies should incorporate sustained attention assessments for all participant groups to allow for a more comprehensive evaluation of attentional processes. Third, the use of cross-sectional data prevents causal inferences about symptom-level relationships. Longitudinal studies are needed to gain insight into temporal relationships and the sequence of item activation. Fourth, network analysis does not account for covariates or confounders, as the partial correlations between items only control for other items in the network. External factors that were not modeled, such as stressors, mood, emotion regulation abilities, and gender, might drive observed associations. A deeper exploration of these factors may provide further insight into how cognitive functions are influenced over time in type 1 diabetes patients. Lastly, the study relied solely on the Wisconsin Card Sorting Test (WCST) to measure cognitive flexibility. While this test is widely used, it may not fully capture the complexity of cognitive flexibility. Future research would benefit from incorporating multiple assessments, such as task-switching paradigms and other neuropsychological tools, to obtain a more comprehensive understanding of cognitive flexibility in this patient population.

## Electronic supplementary material

Below is the link to the electronic supplementary material.

Supplementary Material 1

## Data Availability

The data that support the findings of this study are available from the corresponding author upon reasonable request.
