# Supplementary material for: Cognitive flexibility is associated with the age of onset and duration among patients with type 1 diabetes
Source: Sci Rep. 2025 Jul 7;15:24222. doi: 10.1038/s41598-025-99678-2 (PMC12234857; doi:10.1038/s41598-025-99678-2)
Supplement: Supplementary file 1 — Supplementary Material 1 [file 41598_2025_99678_MOESM1_ESM.docx]

**Supplementary Materials**

**Supplemental Figure**

**
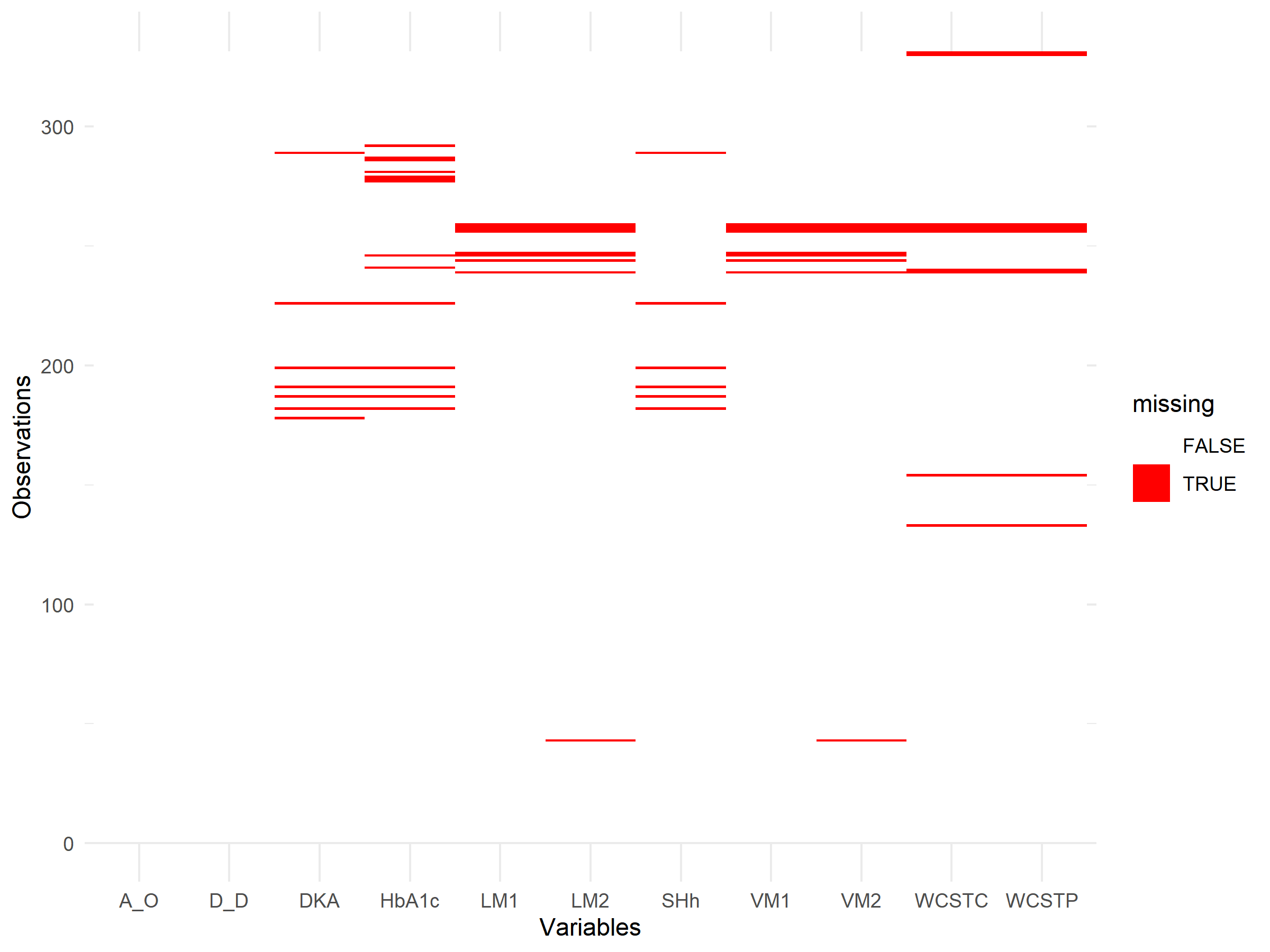
**

**Figure S1.** Visualization of missing values across the entire data set.


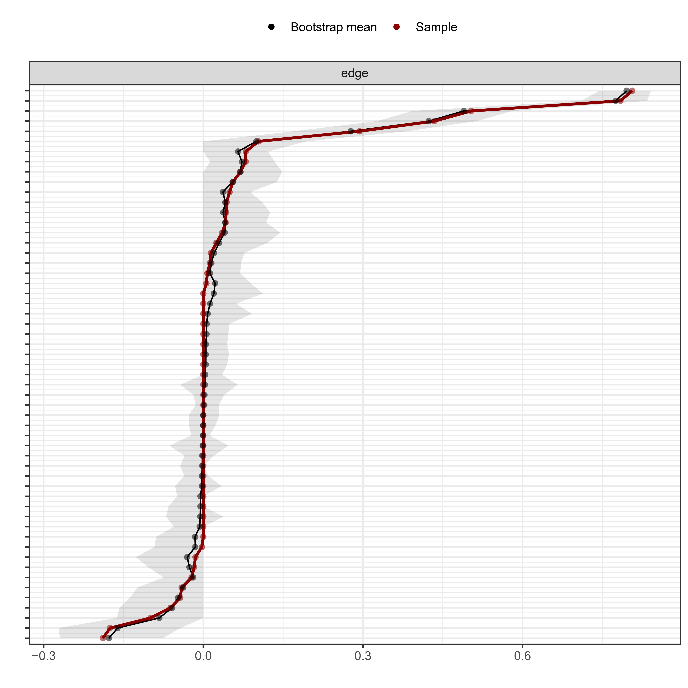


**Figure S2.** Edge-weight accuracy of the cognitive functions and clinical-glycemic characteristics network (N = 331).


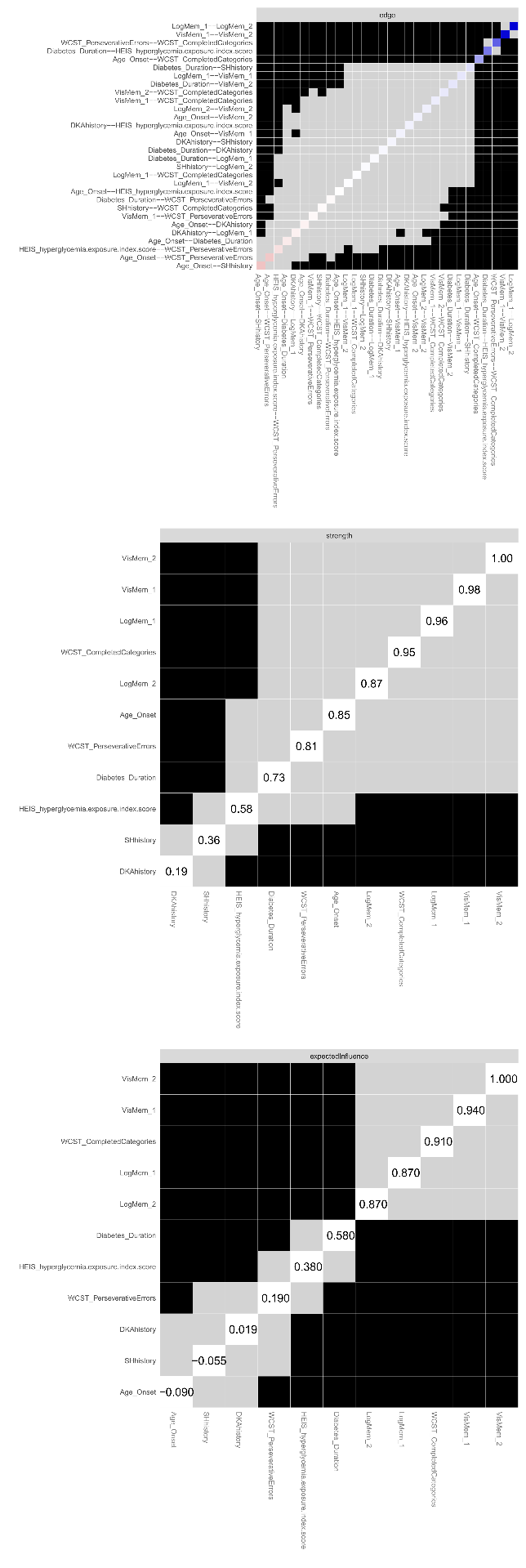


**Figure S3.** Differences in edge-weights and centralities of the cognitive functions and clinical-glycemic characteristics network. Gray boxes indicate nodes or edges that do not differ significantly and black boxes represent nodes or edges that differ significantly.


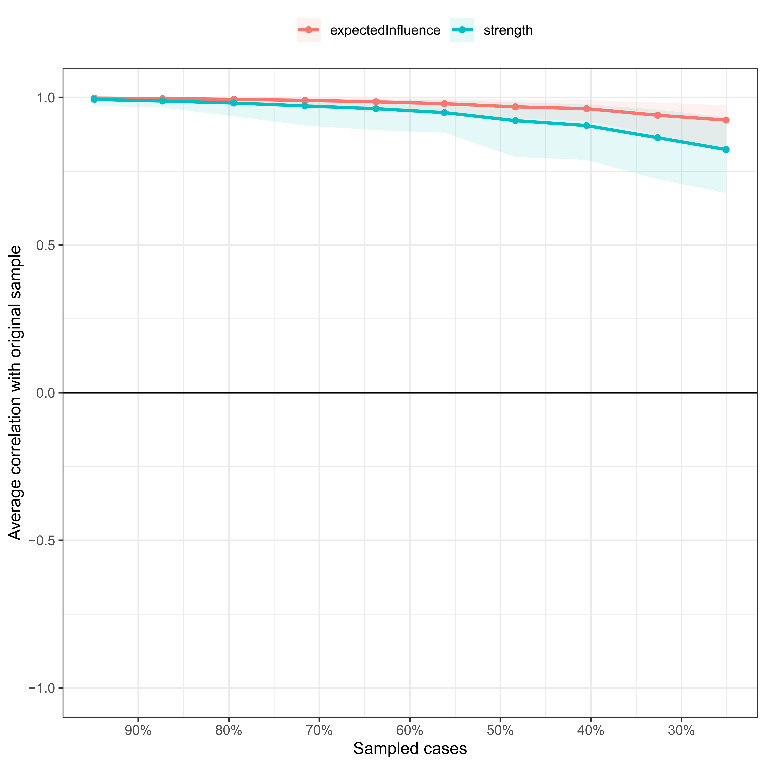


**Figure S4.** Centrality stability of the cognitive functions and clinical-glycemic characteristics network if portions of the participants are excluded.


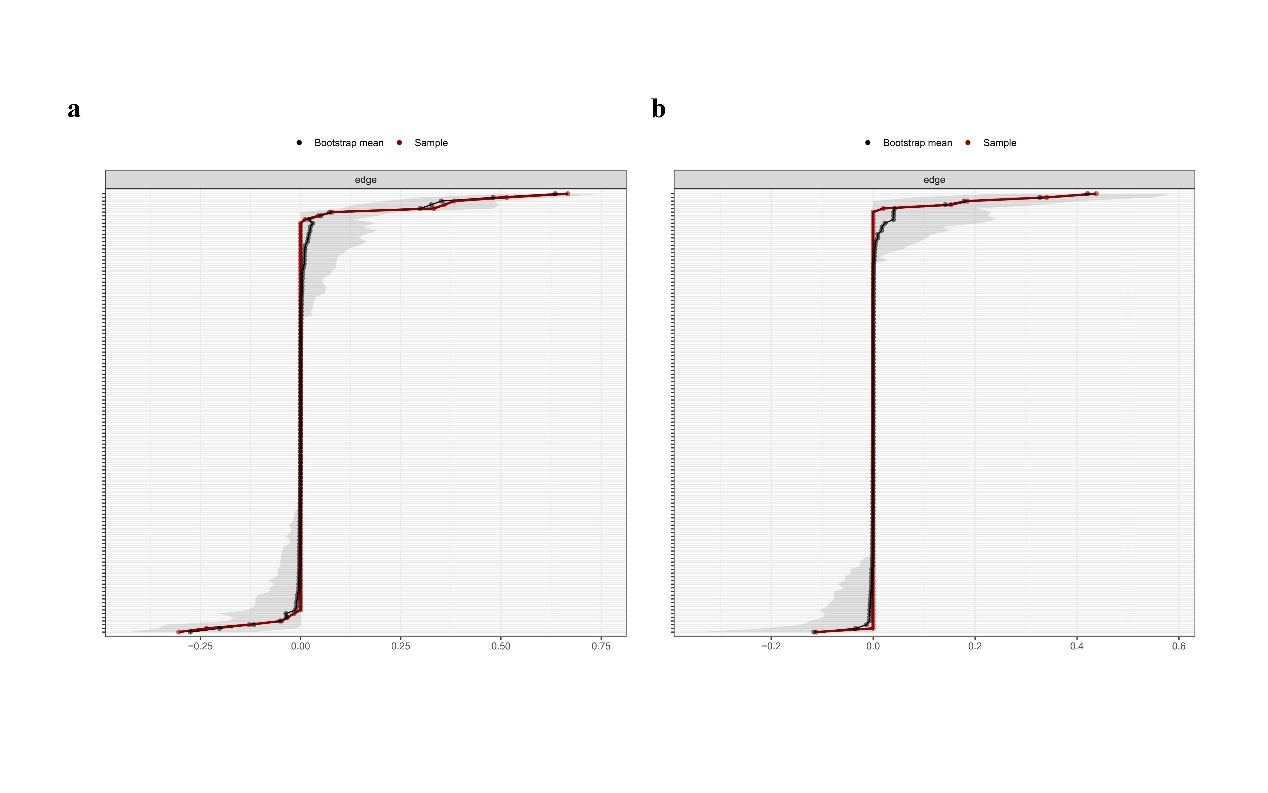


**Figure S5.** Edge-weight accuracy of the cognitive functions and clinical-glycemic characteristics networks for childhood-onset adult patients (n = 78, **a**) and adult-onset adult patients (n = 72, **b**).


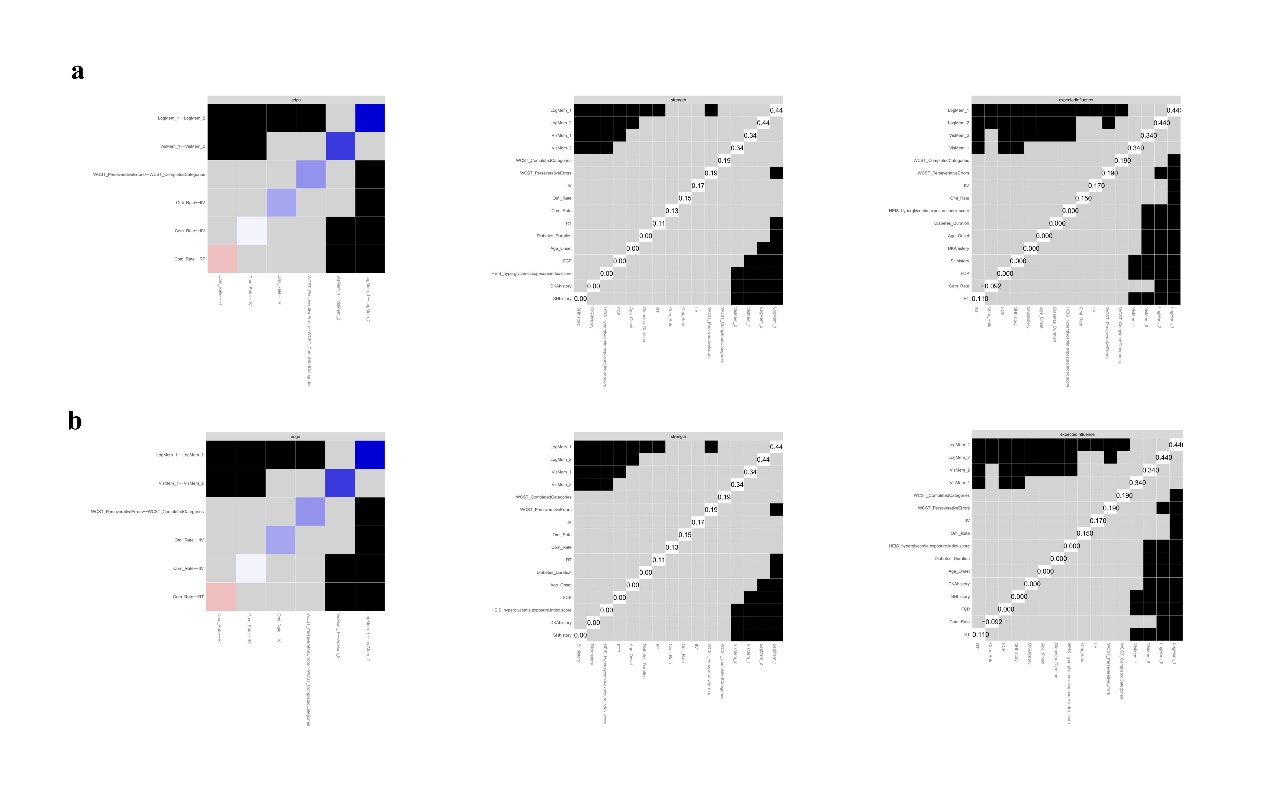


**Figure S6.** Differences in edge-weights and centralities of the cognitive functions and clinical-glycemic characteristics networks for childhood-onset adult patients (**a**) and adult-onset adult patients (**b**). Gray boxes indicate nodes or edges that do not differ significantly and black boxes represent nodes or edges that differ significantly.


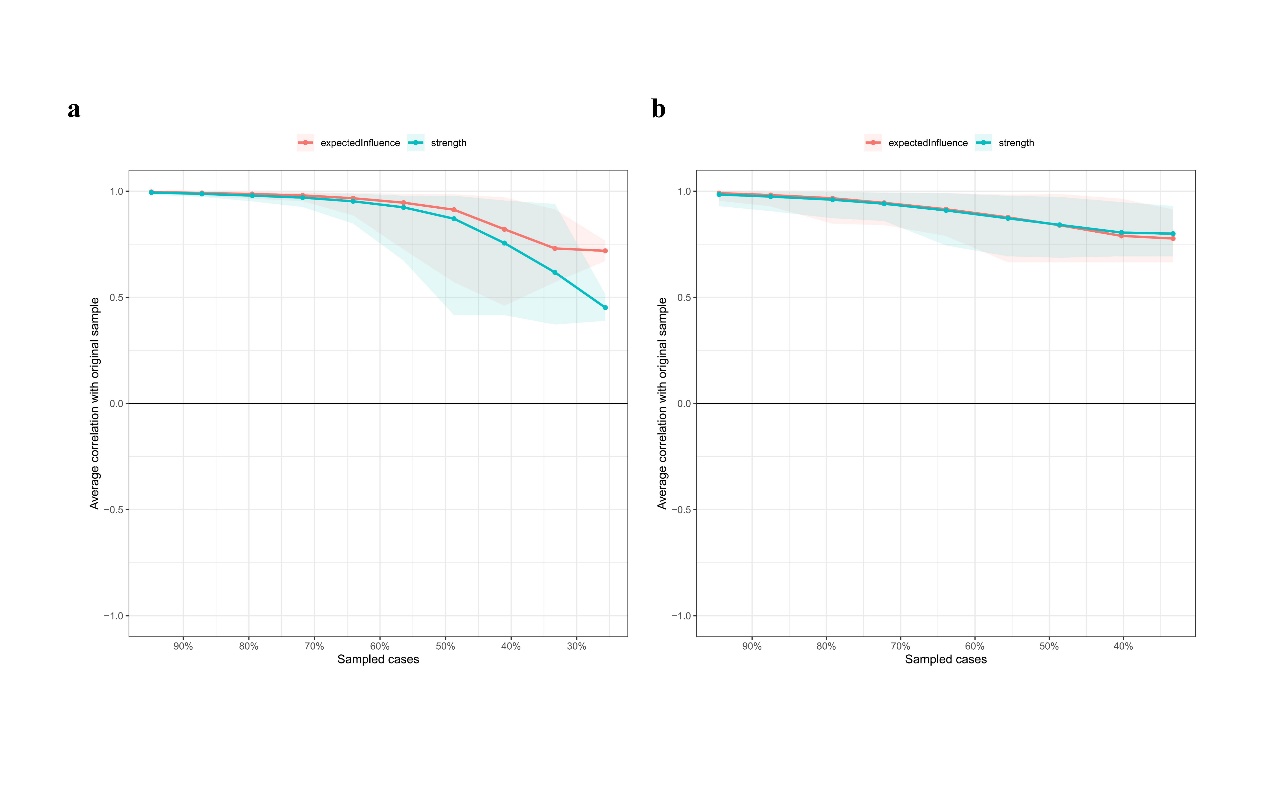


**Figure S7.** Centrality stability of the cognitive functions and clinical-glycemic characteristics networks if portion of the participants are excluded. **a** is for childhood-onset adult patients, and **b** is for adult-onset adult patients.


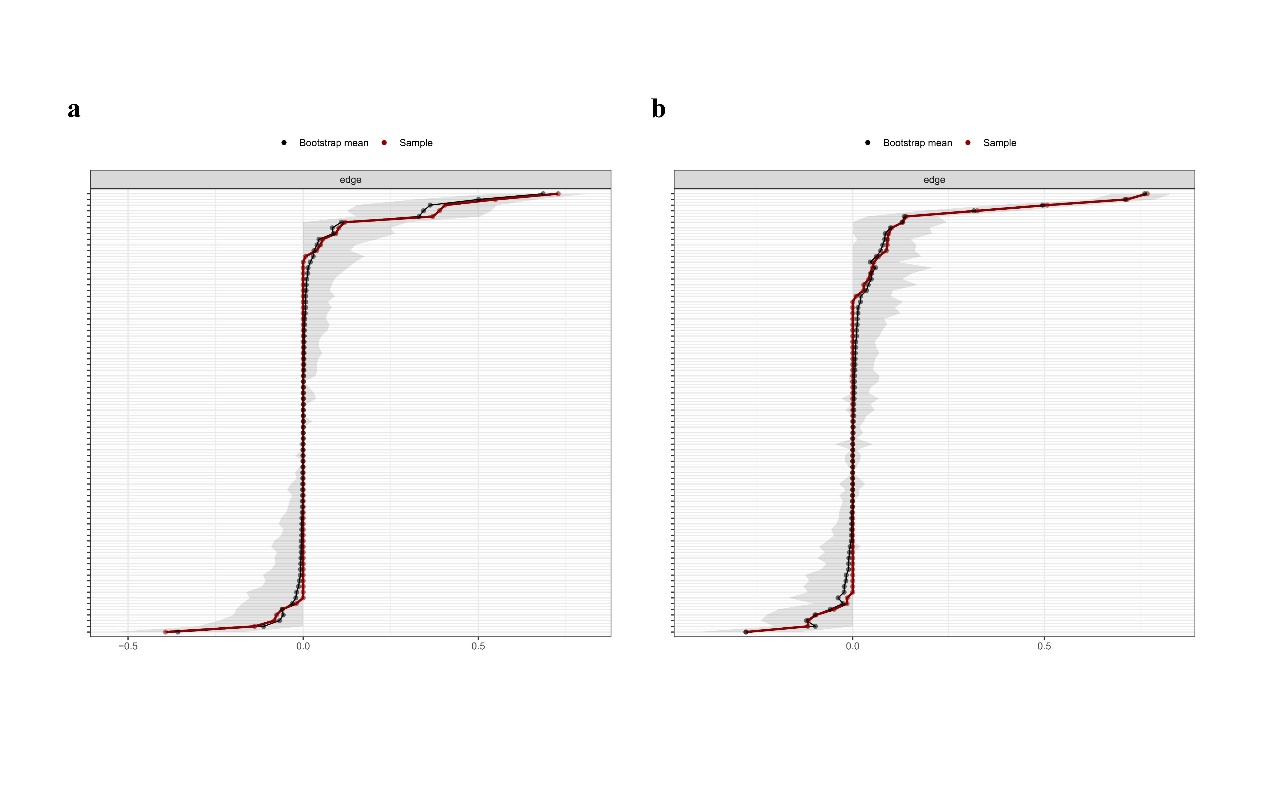


**Figure S8.** Edge-weight accuracy of the cognitive functions and clinical-glycemic characteristics networks of childhood-onset type 1 diabetes for child group (n = 181, **a**) and adult group (n = 78, **b**).


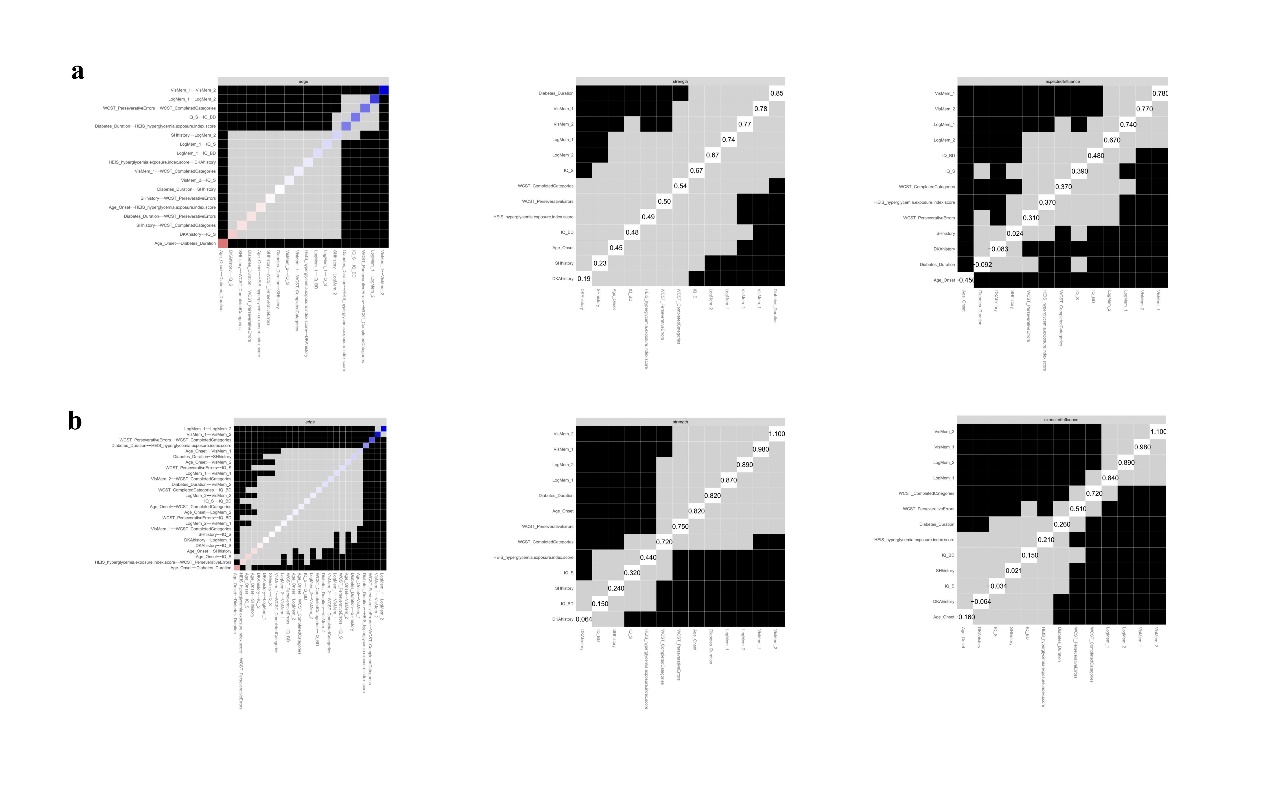


**Figure S9.** Differences of edge-weights and centralities of the cognitive functions and clinical-glycemic characteristics networks of childhood-onset type 1 diabetes for child group (**a**) and adult group (**b**). Gray boxes indicate nodes or edges that do not differ significantly and black boxes represent nodes or edges that differ significantly.


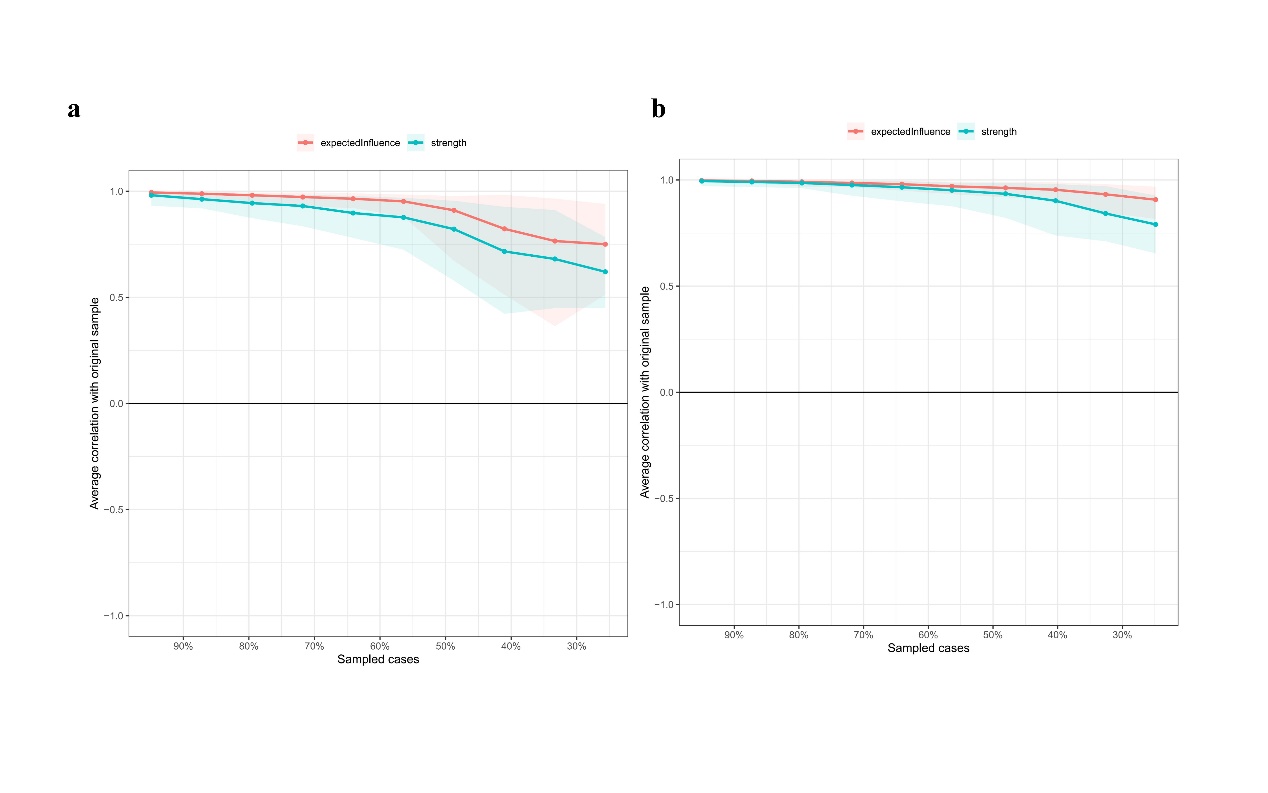


**Figure S10.** Centrality stability of the cognitive functions and clinical-glycemic characteristics networks of childhood-onset type 1 diabetes if portion of the participants are excluded. **a** is for child group, and **b** is for adult group.

**Supplemental Table**

**Table S1.** Details on cognitive testing used

| Cognitive testing | Description |
| --- | --- |
| General Intellectual Ability | We measured general intellectual ability using the Wechsler Intelligence Scale for Children—Chinese Revision for participants under 16 years and the Wechsler Adult Intelligence Scale—Chinese Revision for those 16 years or older. Despite some differences, these scales are highly correlated (>0.9), indicating they measure similar structures.  Subtest scores were combined to calculate IQ using standard methods. The scales were revised into a Chinese version by Gong Yaoxian and others in 1981. In this study, we used five subtests—information, digit span, similarities, picture completion, and block design—to assess IQ. According to the Wechsler Abbreviated Scale of Intelligence, the full-scale IQ score was computed by summing age-scaled subtest scores. |
| The Similarity subtest of the Wechsler Intelligence Scale | The Similarity subtest of the Wechsler Intelligence Scale requires the test taker to choose the option most like a given item, which could be geometric shapes, symbols, or other abstract forms. The test involves scanning items quickly within a time limit and selecting the most similar one. This assesses visual reasoning and abstract thinking abilities. Evaluators use this test to understand how well the test taker processes abstract information and solves problems. Scores are compared to a standardized reference group to determine the level of similarity reasoning ability, with higher scores indicating stronger visual reasoning and abstract thinking skills. |
| The Block Design subtest of the Wechsler Intelligence Scale | The Block Design subtest of the Wechsler Intelligence Scale requires the test taker to recreate a target pattern using blocks. The blocks vary in shape, size, and color. The task involves observing pictures of block arrangements and matching them within a time limit, requiring three-dimensional spatial visualization, pattern recognition, and problem-solving. This subtest assesses spatial perception and processing abilities. Evaluators use the results to understand how well the test taker processes and solves spatial problems. Scores are compared to a standardized reference group, with higher scores indicating stronger spatial visualization and problem-solving skills. |
| Wechsler Memory Scale, WMS | Two tests were selected from the WMS: logical memory and visual memory. For logical memory, the experimenter reads a short story to participants, who must immediately recall it and then recall it again after 30 minutes. The score is the total number of words recalled correctly. For visual memory, participants view two pictures for 10 seconds each and then draw them from memory immediately and after 30 minutes. The score is the total number of pictures recalled correctly. |
| Wisconsin Card Sorting Test, WSCT | In this study, we used the Wisconsin Card Sorting Test-64 Card Version 2000, administered  on a personal computer ^1^.This test measures cognitive flexibility using 4 stimulus cards and 48 test cards. The stimulus cards display different shapes, colors, and quantities (e.g., a red triangle, two yellow stars). The test cards are random combinations of these features. Participants categorize the test cards by matching them to the stimulus cards. The experimenter provides feedback on accuracy but does not reveal the classification rule. After six correct matches, the rule changes (color, shape, number) and participants continue. Measures include the total number of categories completed and perseverative errors (repeating the same mistake after feedback). Perseverative errors are exact repetitions of immediately preceding incorrect responses, and non-perseverative errors include all errors other than perseverative errors. Difficulty maintaining sets is reflected by an incorrect response after two or more consecutive correct responses. Perseverative errors and  the number of completed categories are considered to be important psychometric parameters of the WCST, with lower scores indicating more severe executive function damage ^2^. |
| Sustained Attention to Response Task, SART | The participants were individually seated 50 cm from a computer screen and completed their tests using E-prime 2.0 software (Psychology Software Tools, Pittsburgh, Pennsylvania).  The SART included 225 trials, each displaying a single digit (1–9) for 250 milliseconds, followed by a 900-millisecond mask (a ring with a cross, 2.5 cm diameter) in black bold font on a white background. Participants responded to “Go” targets by pressing the left mouse button quickly, except for the number 3 (“No-go” target, 25 of 225 trials). Each digit appeared in five random font sizes (19, 26, 36, 66, 80 points), repeated five times. A practice session of eight trials with two “No-go” targets (digit “3”) ensured task comprehension.After the task, the following were calculated: omission errors (failures to respond to “Go” targets), commission errors (failures to withhold response to “No-go” targets), mean reaction time (RT) before a correct response, and intraindividual variability (IIV; RT standard deviation/mean RT). |

1 Basso, M. R., Lowery, N., Ghormley, C. & Bornstein, R. A. Practice effects on the Wisconsin Card Sorting Test-64 Card version across 12 months. *Clin Neuropsychol* **15**, 471-478 (2001).

2 Nyhus, E. & Barceló, F. The Wisconsin Card Sorting Test and the cognitive assessment of prefrontal executive functions: a critical update. *Brain Cogn* **71**, 437-451, doi:10.1016/j.bandc.2009.03.005 (2009).

**Table S2.** Zero-order correlation matrix for all the participants (N = 331. Note: * p<0.05 (2-tailed); ** p<0.01 (2-tailed).

|  | A_O | D_D | DKA | SHh | HEI | LM_1 | LM_2 | VM_1 | VM_2 | WCST_P | WCST_C |
| --- | --- | --- | --- | --- | --- | --- | --- | --- | --- | --- | --- |
| A_O | 1 |  |  |  |  |  |  |  |  |  |  |
| D_D | -0.152** | 1 |  |  |  |  |  |  |  |  |  |
| DKA | -0.146** | 0.139* | 1 |  |  |  |  |  |  |  |  |
| SHh | -0.261** | 0.058 | 0.046 | 1 |  |  |  |  |  |  |  |
| HEI | -0.095 | 0.660** | 0.114* | -0.009 | 1 |  |  |  |  |  |  |
| LM_1 | 0.039 | 0.096 | -0.139* | -0.008 | -0.005 | 1 |  |  |  |  |  |
| LM_2 | -0.025 | 0.081 | -0.052 | 0.018 | 0.038 | 0.872** | 1 |  |  |  |  |
| VM_1 | 0.169** | 0.160** | -0.049 | -0.076 | 0.077 | 0.384** | 0.380** | 1 |  |  |  |
| VM_2 | 0.162** | 0.205** | -0.042 | -0.076 | 0.086 | 0.369** | 0.371** | 0.896** | 1 |  |  |
| WCST_P | -0.120* | -0.159** | -0.041 | 0.041 | -0.193** | 0.085 | 0.052 | -0.015 | -0.004 | 1 |  |
| WCST_C | 0.271** | -0.002 | -0.068 | -0.109 | -0.065 | 0.158** | 0.084 | 0.269** | 0.264** | 0.508** | 1 |

**Table S3.** Zero-order correlation matrix for childhood-onset adult patients (N = 78. Note: * p<0.05 (2-tailed); ** p<0.01 (2-tailed).

|  | A_O | D_D | HEI | DKA |  | SHh | FCP | LM_1 | LM_2 | VM_1 | VM_2 | WCST_P | WCST_C | Omi_R | Com_R | RT | IIV |
| --- | --- | --- | --- | --- | --- | --- | --- | --- | --- | --- | --- | --- | --- | --- | --- | --- | --- |
| A_O | 1 |  |  |  |  |  |  |  |  |  |  |  |  |  |  |  |  |
| D_D | -0.646** | 1 |  |  |  |  |  |  |  |  |  |  |  |  |  |  |  |
| HEI | -0.489** | 0.733** | 1 |  |  |  |  |  |  |  |  |  |  |  |  |  |  |
| DKA | -0.119 | 0.196 | 0.242* | 1 |  |  |  |  |  |  |  |  |  |  |  |  |  |
| SHh | -0.161 | 0.160 | 0.070 | 0.049 |  | 1 |  |  |  |  |  |  |  |  |  |  |  |
| FCP | 0.246* | -0.417** | -0.134 | -0.239* |  | -0.154 | 1 |  |  |  |  |  |  |  |  |  |  |
| LM_1 | -0.040 | -0.102 | -0.147 | -0.154 |  | 0.161 | -0.030 | 1 |  |  |  |  |  |  |  |  |  |
| LM_2 | -0.028 | -0.117 | -0.124 | -0.137 |  | 0.305** | -0.051 | 0.862** | 1 |  |  |  |  |  |  |  |  |
| VM_1 | -0.027 | 0.056 | 0.000 | -0.074 |  | 0.092 | -0.040 | 0.110 | 0.133 | 1 |  |  |  |  |  |  |  |
| VM_2 | -0.057 | 0.074 | 0.023 | -0.103 |  | 0.153 | -0.021 | 0.080 | 0.145 | 0.949** | 1 |  |  |  |  |  |  |
| WCST_P | 0.135 | -0.299** | -0.213 | 0.012 |  | -0.269** | 0.218 | -0.009 | -0.037 | -0.036 | -0.098 | 1 |  |  |  |  |  |
| WCST_C | 0.105 | -0.112 | -0.065 | -0.049 |  | -0.300** | 0.169 | -0.029 | -0.042 | 0.169 | 0.106 | 0.635** | 1 |  |  |  |  |
| Omi_R | 0.239* | -0.030 | 0.011 | 0.117 |  | 0.282* | -0.005 | -0.089 | -0.049 | 0.188 | 0.212 | -0.029 | -0.016 | 1 |  |  |  |
| Com_R | -0.018 | -0.009 | 0.013 | 0.092 |  | 0.250* | 0.005 | -0.066 | 0.056 | 0.077 | 0.097 | -0.001 | -0.061 | 0.448** | 1 |  |  |
| RT | 0.007 | -0.013 | 0.065 | -0.044 |  | 0.103 | 0.104 | 0.014 | -0.020 | -0.105 | -0.060 | -0.118 | -0.032 | 0.280* | -0.316** | 1 |  |
| IIV | 0.026 | 0.038 | 0.133 | -0.117 |  | 0.028 | 0.050 | -0.158 | -0.143 | -0.022 | -0.045 | -0.099 | -0.044 | 0.275* | 0.118 | 0.206 | 1 |

**Table S4.** Zero-order correlation matrix for adult-onset adult patients (N = 72. Note: * p<0.05 (2-tailed); ** p<0.01 (2-tailed).

|  | A_O | D_D | HEI | DKA | SHh | FCP | LM_1 | LM_2 | VM_1 | VM_2 | WCST_P | WCST_C | Omi_R | Com_R | RT | IIV |
| --- | --- | --- | --- | --- | --- | --- | --- | --- | --- | --- | --- | --- | --- | --- | --- | --- |
| A_O | 1 |  |  |  |  |  |  |  |  |  |  |  |  |  |  |  |
| D_D | -0.042 | 1 |  |  |  |  |  |  |  |  |  |  |  |  |  |  |
| HEI | 0.064 | 0.431** | 1 |  |  |  |  |  |  |  |  |  |  |  |  |  |
| DKA | -0.115 | 0.377** | 0.232* | 1 |  |  |  |  |  |  |  |  |  |  |  |  |
| SHh | -0.017 | 0.111 | -0.032 | 0.148 | 1 |  |  |  |  |  |  |  |  |  |  |  |
| FCP | 0.219 | -0.308** | -0.255* | -0.082 | -0.202 | 1 |  |  |  |  |  |  |  |  |  |  |
| LM_1 | -0.271* | -0.003 | -0.169 | -0.155 | 0.107 | -0.024 | 1 |  |  |  |  |  |  |  |  |  |
| LM_2 | -0.272* | -0.072 | -0.065 | -0.086 | 0.027 | -0.046 | 0.859** | 1 |  |  |  |  |  |  |  |  |
| VM_1 | -0.283* | -0.060 | -0.144 | -0.019 | -0.071 | 0.037 | 0.203 | 0.166 | 1 |  |  |  |  |  |  |  |
| VM_2 | -0.195 | -0.006 | -0.187 | -0.055 | -0.121 | 0.047 | 0.289* | 0.253* | 0.770** | 1 |  |  |  |  |  |  |
| WCST_P | -0.097 | 0.214 | 0.137 | 0.089 | 0.048 | -0.275* | 0.193 | 0.137 | 0.032 | 0.122 | 1 |  |  |  |  |  |
| WCST_C | -0.123 | 0.059 | -0.017 | 0.029 | -0.006 | -0.163 | 0.294* | 0.228 | 0.351** | 0.296* | 0.660** | 1 |  |  |  |  |
| Omi_R | -0.040 | 0.115 | 0.097 | 0.172 | 0.311** | -0.085 | -0.221 | -0.180 | -0.072 | -0.038 | -0.133 | -0.297* | 1 |  |  |  |
| Com_R | -0.134 | -0.018 | -0.110 | -0.051 | 0.092 | 0.150 | -0.063 | -0.055 | -0.017 | -0.006 | 0.069 | -0.172 | 0.678** | 1 |  |  |
| RT | 0.296* | 0.109 | 0.074 | 0.048 | 0.060 | 0.028 | -0.083 | -0.068 | -0.291* | -0.208 | -0.211 | -0.226 | -0.015 | -0.223 | 1 |  |
| IIV | 0.059 | -0.051 | -0.159 | -0.094 | -0.069 | 0.102 | -0.106 | -0.097 | -0.304** | -0.206 | -0.084 | -0.221 | 0.115 | 0.162 | 0.061 | 1 |

**Table S5.** Zero-order correlation matrix for childhood-onset child patients (N = 181. Note: * p<0.05 (2-tailed); ** p<0.01 (2-tailed).

|  | A_O | D_D | HEI | DKA | SHh | LM_1 | LM_2 | VM_1 | VM_2 | WCST_P | WCST_C | IQ_S | IQ_B |
| --- | --- | --- | --- | --- | --- | --- | --- | --- | --- | --- | --- | --- | --- |
| A_O | 1 |  |  |  |  |  |  |  |  |  |  |  |  |
| D_D | -0.495** | 1 |  |  |  |  |  |  |  |  |  |  |  |
| HEI | -0.200** | 0.619** | 1 |  |  |  |  |  |  |  |  |  |  |
| DKA | -0.028 | 0.154* | 0.087 | 1 |  |  |  |  |  |  |  |  |  |
| SHh | -0.239** | 0.198** | 0.080 | -0.003 | 1 |  |  |  |  |  |  |  |  |
| LM_1 | 0.269** | 0.081 | 0.007 | -0.131 | -0.018 | 1 |  |  |  |  |  |  |  |
| LM_2 | 0.270** | 0.129 | 0.076 | -0.038 | -0.022 | 0.884** | 1 |  |  |  |  |  |  |
| VM_1 | 0.447** | 0.085 | 0.036 | -0.030 | -0.048 | 0.485** | 0.502** | 1 |  |  |  |  |  |
| VM_2 | 0.417** | 0.088 | 0.019 | -0.012 | -0.046 | 0.440** | 0.462** | 0.893** | 1 |  |  |  |  |
| WCST_P | -0.008 | -0.125 | -0.246** | -0.093 | 0.031 | 0.110 | 0.069 | 0.030 | 0.064 | 1 |  |  |  |
| WCST_C | 0.198** | -0.107 | -0.185* | -0.046 | -0.006 | 0.148* | 0.096 | 0.231** | 0.262** | 0.595** | 1 |  |  |
| IQ_S | -0.240** | -0.006 | -0.078 | -0.120 | -0.077 | 0.064 | 0.049 | -0.137 | -0.136 | 0.161* | 0.059 | 1 |  |
| IQ_B | -0.078 | -0.008 | -0.120 | 0.043 | -0.017 | 0.024 | 0.035 | 0.055 | 0.045 | 0.217** | 0.204** | 0.128 | 1 |

**Table S6.** Zero-order correlation matrix for childhood-onset adult patients (N = 78. Note: * p<0.05 (2-tailed); ** p<0.01 (2-tailed). (in second networks comparison)

|  | A_O | D_D | HEI | DKA | SHh | LM_1 | LM_2 | VM_1 | VM_2 | WCST_P | WCST_C | IQ_S | IQ_B |
| --- | --- | --- | --- | --- | --- | --- | --- | --- | --- | --- | --- | --- | --- |
| A_O | 1 |  |  |  |  |  |  |  |  |  |  |  |  |
| D_D | -0.646** | 1 |  |  |  |  |  |  |  |  |  |  |  |
| HEI | -0.445** | 0.657** | 1 |  |  |  |  |  |  |  |  |  |  |
| DKA | -0.101 | 0.203 | 0.234* | 1 |  |  |  |  |  |  |  |  |  |
| SHh | -0.175 | 0.171 | 0.060 | 0.052 | 1 |  |  |  |  |  |  |  |  |
| LM_1 | -0.005 | -0.101 | -0.147 | -0.137 | 0.120 | 1 |  |  |  |  |  |  |  |
| LM_2 | -0.013 | -0.124 | -0.138 | -0.153 | 0.297** | 0.858** | 1 |  |  |  |  |  |  |
| VM_1 | -0.012 | 0.068 | 0.005 | -0.065 | 0.061 | 0.088 | 0.115 | 1 |  |  |  |  |  |
| VM_2 | -0.045 | 0.089 | 0.031 | -0.105 | 0.128 | 0.056 | 0.127 | 0.948** | 1 |  |  |  |  |
| WCST_P | 0.129 | -0.283* | -0.175 | 0.015 | -0.277* | -0.021 | -0.045 | -0.040 | -0.105 | 1 |  |  |  |
| WCST_C | 0.108 | -0.100 | -0.057 | -0.013 | -0.307** | -0.052 | -0.064 | 0.160 | 0.098 | 0.637** | 1 |  |  |
| IQ_S | 0.016 | -0.163 | -0.136 | -0.337** | -0.259* | 0.437** | 0.296** | 0.173 | 0.180 | 0.063 | 0.067 | 1 |  |
| IQ_B | 0.081 | -0.058 | -0.152 | -0.065 | -0.244* | 0.283* | 0.084 | 0.051 | 0.047 | 0.043 | 0.139 | 0.677** | 1 |

**Table S7.** Edge weights matrix of the cognitive functions and clinical-glycemic characteristics network for all the participants (N = 331).

|  | A_O | D_D | DKA | SHh | HEI | LM_1 | LM_2 | VM_1 | VM_2 | WCST_P | WCST_C |
| --- | --- | --- | --- | --- | --- | --- | --- | --- | --- | --- | --- |
| A_O | 0 | -0.112 | -0.089 | -0.204 | 0 | 0 | -0.027 | 0.028 | 0.026 | -0.229 | 0.280 |
| D_D | -0.112 | 0 | 0.065 | 0 | 0.604 | 0.007 | 0 | 0 | 0.076 | -0.055 | 0.020 |
| DKA | -0.089 | 0.065 | 0 | 0 | 0.018 | -0.059 | 0 | 0 | 0 | 0 | 0 |
| SHh | -0.204 | 0 | 0 | 0 | 0 | 0 | 0 | 0 | -0.004 | 0 | -0.016 |
| HEI | 0 | 0.604 | 0.018 | 0 | 0 | 0 | 0 | 0 | 0 | -0.088 | 0 |
| LM_1 | 0 | 0.007 | -0.059 | 0 | 0 | 0 | 0.811 | 0.057 | 0.004 | 0.012 | 0.018 |
| LM_2 | -0.027 | 0.000 | 0 | 0 | 0 | 0.811 | 0 | 0.021 | 0.044 | 0 | 0 |
| VM_1 | 0.028 | 0 | 0 | 0 | 0 | 0.057 | 0.021 | 0 | 0.827 | -0.037 | 0.071 |
| VM_2 | 0.026 | 0.076 | 0 | -0.004 | 0 | 0.004 | 0.044 | 0.827 | 0 | 0 | 0.045 |
| WCST_P | -0.229 | -0.055 | 0 | 0 | -0.088 | 0.012 | 0 | -0.037 | 0 | 0 | 0.507 |
| WCST_C | 0.280 | 0.020 | 0 | -0.016 | 0 | 0.018 | 0 | 0.071 | 0.045 | 0.507 | 0 |

**Table S8.** Overview of the edges between specific cognitive functions domains and clinical-glycemic characteristics in the network for all the participants (N =331). The total strength in the table is the absolute value.

|  | | | | | | |
| --- | --- | --- | --- | --- | --- | --- |
|  | Total edges | Positive edges | Negative edges | Total strength | Positive strength | Negative strength |
| All edges | 31 | 20 | 11 | 4.460 | 3.539.56 | -0.921 |
| Between cognitive functions nodes | 12 | 11 | 1 | 2.453 | 2.416 | -0.037 |
| Between clinical-glycemic nodes | 6 | 3 | 3 | 1.092 | 0.687 | -0.405 |
| Interaction between cognitive functions and clinical-glycemic characteristics | 13 | 6 | 7 | 0.915 | 0.437 | -0.478 |
|  |  |  |  |  |  |  |

**Table S9.** Edge weights matrix of the cognitive functions and clinical-glycemic network for childhood-onset adult patients (N = 78).

|  | A_O | D_D | HEI | DKA | SHh | FCP | LM_1 | LM_2 | VM_1 | VM_2 | WCST_P | WCST_C | Omi_R | Com_R | RT | IIV |
| --- | --- | --- | --- | --- | --- | --- | --- | --- | --- | --- | --- | --- | --- | --- | --- | --- |
| A_O | 0 | -0.369 | -0.070 | 0 | 0 | 0 | 0 | 0 | 0 | 0 | 0 | 0 | 0.058 | 0 | 0 | 0 |
| D_D | -0.369 | 0 | 0.481 | 0 | 0 | -0.188 | 0 | 0 | 0 | 0 | -0.084 | 0 | 0 | 0 | 0 | 0 |
| HEI | -0.070 | 0.481 | 0 | 0.053 | 0 | 0 | 0 | 0 | 0 | 0 | 0 | 0 | 0 | 0 | 0 | 0 |
| DKA | 0 | 0 | 0.053 | 0 | 0 | -0.058 | 0 | 0 | 0 | 0 | 0 | 0 | 0 | 0 | 0 | 0 |
| SHh | 0 | 0 | 0 | 0 | 0 | 0 | 0 | 0.096 | 0 | 0 | -0.043 | -0.094 | 0.091 | 0.050 | 0 | 0 |
| FCP | 0 | -0.188 | 0 | -0.058 | 0 | 0 | 0 | 0 | 0 | 0 | 0.015 | 0 | 0 | 0 | 0 | 0 |
| LM_1 | 0 | 0 | 0 | 0 | 0 | 0 | 0 | 0.689 | 0 | 0 | 0 | 0 | 0 | 0 | 0 | 0 |
| LM_2 | 0 | 0 | 0 | 0 | 0.096 | 0 | 0.689 | 0 | 0 | 0 | 0 | 0 | 0 | 0 | 0 | 0 |
| VM_1 | 0 | 0 | 0 | 0 | 0 | 0 | 0 | 0 | 0 | 0.779 | 0 | 0 | 0 | 0 | 0 | 0 |
| VM_2 | 0 | 0 | 0 | 0 | 0 | 0 | 0 | 0 | 0.779 | 0 | 0 | 0 | 0.025 | 0 | 0 | 0 |
| WCST_P | 0 | -0.084 | 0 | 0 | -0.043 | 0.015 | 0 | 0 | 0 | 0 | 0 | 0.455 | 0 | 0 | 0 | 0 |
| WCST_C | 0 | 0 | 0 | 0 | -0.094 | 0 | 0 | 0 | 0 | 0 | 0.455 | 0 | 0 | 0 | 0 | 0 |
| Omi_R | 0.058 | 0 | 0 | 0 | 0.091 | 0 | 0 | 0 | 0 | 0.025 | 0 | 0 | 0 | 0.291 | 0.155 | 0.097 |
| Com_R | 0 | 0 | 0 | 0 | 0.050 | 0 | 0 | 0 | 0 | 0 | 0 | 0 | 0.291 | 0 | -0.185 | 0 |
| RT | 0 | 0 | 0 | 0 | 0 | 0 | 0 | 0 | 0 | 0 | 0 | 0 | 0.155 | -0.185 | 0 | 0.024 |
| IIV | 0 | 0 | 0 | 0 | 0 | 0 | 0 | 0 | 0 | 0 | 0 | 0 | 0.097 | 0 | 0.024 | 0 |

**Table S10.** Overview of the edges between specific cognitive functions domains and clinical-glycemic characteristics in the network for childhood-onset adult patients (N =78). The total strength in the table is the absolute value.

|  | | | | | | |
| --- | --- | --- | --- | --- | --- | --- |
|  | Total edges | Positive edges | Negative edges | Total strength | Positive strength | Negative strength |
| All edges | 23 | 15 | 8 | 4.450 | 3.359 | -1.091 |
| Between cognitive functions nodes | 9 | 8 | 1 | 2.700 | 2.515 | -0.185 |
| Between clinical-glycemic nodes | 6 | 2 | 4 | 1.219 | 0.534 | -0.685 |
| Interaction between cognitive functions and clinical-glycemic characteristics | 8 | 5 | 3 | 0.530 | 0.309 | -0.221 |
|  |  |  |  |  |  |  |

**Table S11.** Edge weights matrix of the cognitive functions and clinical-glycemic network for adult-onset adult patients (N = 72).

|  | A_O | D_D | HEI | DKA | SHh | FCP | LM_1 | LM_2 | VM_1 | VM_2 | WCST_P | WCST_C | Omi_R | Com_R | RT | IIV |
| --- | --- | --- | --- | --- | --- | --- | --- | --- | --- | --- | --- | --- | --- | --- | --- | --- |
| A_O | 0 | 0 | 0 | 0 | 0 | 0 | 0 | 0 | 0 | 0 | 0 | 0 | 0 | 0 | 0 | 0 |
| D_D | 0 | 0 | 0.107 | 0.053 | 0 | 0 | 0 | 0 | 0 | 0 | 0 | 0 | 0 | 0 | 0 | 0 |
| HEI | 0 | 0.107 | 0 | 0 | 0 | 0 | 0 | 0 | 0 | 0 | 0 | 0 | 0 | 0 | 0 | 0 |
| DKA | 0 | 0.053 | 0 | 0 | 0 | 0 | 0 | 0 | 0 | 0 | 0 | 0 | 0 | 0 | 0 | 0 |
| SHh | 0 | 0 | 0 | 0 | 0 | 0 | 0 | 0 | 0 | 0 | 0 | 0 | 0 | 0 | 0 | 0 |
| FCP | 0 | 0 | 0 | 0 | 0 | 0 | 0 | 0 | 0 | 0 | 0 | 0 | 0 | 0 | 0 | 0 |
| LM_1 | 0 | 0 | 0 | 0 | 0 | 0 | 0 | 0.536 | 0 | 0 | 0 | 0 | 0 | 0 | 0 | 0 |
| LM_2 | 0 | 0 | 0 | 0 | 0 | 0 | 0.536 | 0 | 0 | 0 | 0 | 0 | 0 | 0 | 0 | 0 |
| VM_1 | 0 | 0 | 0 | 0 | 0 | 0 | 0 | 0 | 0 | 0.446 | 0 | 0.023 | 0 | 0 | 0 | 0 |
| VM_2 | 0 | 0 | 0 | 0 | 0 | 0 | 0 | 0 | 0.446 | 0 | 0 | 0 | 0 | 0 | 0 | 0 |
| WCST_P | 0 | 0 | 0 | 0 | 0 | 0 | 0 | 0 | 0 | 0 | 0 | 0.336 | 0 | 0 | 0 | 0 |
| WCST_C | 0 | 0 | 0 | 0 | 0 | 0 | 0 | 0 | 0.023 | 0 | 0.336 | 0 | 0 | 0 | 0 | 0 |
| Omi_R | 0 | 0 | 0 | 0 | 0 | 0 | 0 | 0 | 0 | 0 | 0 | 0 | 0 | 0.355 | 0 | 0 |
| Com_R | 0 | 0 | 0 | 0 | 0 | 0 | 0 | 0 | 0 | 0 | 0 | 0 | 0.355 | 0 | 0 | 0 |
| RT | 0 | 0 | 0 | 0 | 0 | 0 | 0 | 0 | 0 | 0 | 0 | 0 | 0 | 0 | 0 | 0 |
| IIV | 0 | 0 | 0 | 0 | 0 | 0 | 0 | 0 | 0 | 0 | 0 | 0 | 0 | 0 | 0 | 0 |

**Table S12.** Overview of the edges between specific cognitive functions domains and clinical-glycemic characteristics in the network for adult-onset adult patients (N =72). The total strength in the table is the absolute value.

|  | | | | | | |
| --- | --- | --- | --- | --- | --- | --- |
|  | Total edges | Positive edges | Negative edges | Total strength | Positive strength | Negative strength |
| All edges | 7 | 7 | 0 | 1.856 | 1.856 | 0 |
| Between cognitive functions nodes | 5 | 5 | 0 | 1.696 | 1.696 | 0 |
| Between clinical-glycemic nodes | 2 | 2 | 0 | 0.160 | 0.160 | 0 |
| Interaction between cognitive functions and clinical-glycemic characteristics | 0 | 0 | 0 | 0 | 0 | 0 |
|  |  |  |  |  |  |  |

**Table S13.** Edge weights matrix of the cognitive functions and clinical-glycemic network for childhood-onset child patients (N = 181).

|  | A_O | D_D | HEI | DKA | SHh | LM_1 | LM_2 | VM_1 | VM_2 | WCST_P | WCST_C | IQ_S | IQ_B |
| --- | --- | --- | --- | --- | --- | --- | --- | --- | --- | --- | --- | --- | --- |
| A_O | 0 | -0.416 | 0 | 0 | -0.128 | 0.026 | 0.038 | 0.173 | 0.071 | -0.046 | 0.065 | -0.168 | -0.034 |
| D_D | -0.416 | 0 | 0.502 | 0.078 | 0.056 | 0 | 0.059 | 0.069 | 0.046 | 0 | 0 | 0 | 0 |
| HEI | 0 | 0.502 | 0 | 0 | 0 | 0 | 0 | 0 | 0 | -0.128 | -0.012 | -0.038 | -0.036 |
| DKA | 0 | 0.078 | 0 | 0 | 0 | -0.045 | 0 | 0 | 0 | -0.014 | 0 | -0.066 | 0 |
| SHh | -0.128 | 0.056 | 0 | 0 | 0 | 0 | 0 | 0 | 0 | 0 | 0 | -0.055 | 0 |
| LM_1 | 0.026 | 0 | 0 | -0.045 | 0 | 0 | 0.785 | 0.065 | 0 | 0.011 | 0 | 0.034 | 0 |
| LM_2 | 0.038 | 0.059 | 0 | 0 | 0 | 0.785 | 0 | 0.067 | 0.036 | 0 | 0 | 0 | 0 |
| VM_1 | 0.173 | 0.069 | 0 | 0 | 0 | 0.065 | 0.067 | 0 | 0.767 | 0 | 0 | -0.005 | 0 |
| VM_2 | 0.071 | 0.046 | 0 | 0 | 0 | 0 | 0.036 | 0.767 | 0 | 0 | 0.089 | -0.019 | 0 |
| WCST_P | -0.046 | 0 | -0.128 | -0.014 | 0 | 0.011 | 0 | 0 | 0 | 0 | 0.514 | 0.082 | 0.084 |
| WCST_C | 0.065 | 0 | -0.012 | 0 | 0 | 0 | 0 | 0 | 0.089 | 0.514 | 0 | 0 | 0.078 |
| IQ_S | -0.168 | 0 | -0.038 | -0.066 | -0.055 | 0.034 | 0 | -0.005 | -0.019 | 0.082 | 0 | 0 | 0.050 |
| IQ_B | -0.034 | 0 | -0.036 | 0 | 0 | 0 | 0 | 0 | 0 | 0.084 | 0.078 | 0.050 | 0 |

**Table S14.** Overview of the edges between specific cognitive functions domains and clinical-glycemic characteristics in the network for childhood-onset child patients (N =181. The total strength in the table is the absolute value.

|  | | | | | | |
| --- | --- | --- | --- | --- | --- | --- |
|  | Total edges | Positive edges | Negative edges | Total strength | Positive strength | Negative strength |
| All edges | 39 | 24 | 15 | 5.053 | 3.845 | -1.209 |
| Between cognitive functions nodes | 15 | 13 | 2 | 2.686 | 2.662 | -0.024 |
| Between clinical-glycemic nodes | 5 | 3 | 2 | 1.184 | 0.636 | -0.544 |
| Interaction between cognitive functions and clinical-glycemic characteristics | 19 | 8 | 11 | 1.872 | 0.547 | -0.641 |
|  |  |  |  |  |  |  |

**Table S15.** Edge weights matrix of the cognitive functions and clinical-glycemic network for childhood-onset adult patients (N = 78)（NCT2）.

|  | A_O | D_D | HEI | DKA | SHh | LM_1 | LM_2 | VM_1 | VM_2 | WCST_P | WCST_C | IQ_S | IQ_B |
| --- | --- | --- | --- | --- | --- | --- | --- | --- | --- | --- | --- | --- | --- |
| A_O | 0 | -0.395 | -0.055 | 0 | 0 | 0 | 0 | 0 | 0 | 0 | 0 | 0 | 0 |
| D_D | -0.395 | 0 | 0.408 | 0 | 0 | 0 | 0 | 0 | 0 | -0.070 | 0 | 0 | 0 |
| HEI | -0.055 | 0.408 | 0 | 0.042 | 0 | 0 | 0 | 0 | 0 | 0 | 0 | 0 | 0 |
| DKA | 0 | 0 | 0.042 | 0 | 0 | 0 | 0 | 0 | 0 | 0 | 0 | -0.128 | 0 |
| SHh | 0 | 0 | 0 | 0 | 0 | 0 | 0.093 | 0 | 0 | -0.040 | -0.089 | -0.067 | -0.025 |
| LM_1 | 0 | 0 | 0 | 0 | 0 | 0 | 0.658 | 0 | 0 | 0 | 0 | 0.169 | 0 |
| LM_2 | 0 | 0 | 0 | 0 | 0.093 | 0.658 | 0 | 0 | 0 | 0 | 0 | 0 | 0 |
| VM_1 | 0 | 0 | 0 | 0 | 0 | 0 | 0 | 0 | 0.762 | 0 | 0 | 0 | 0 |
| VM_2 | 0 | 0 | 0 | 0 | 0 | 0 | 0 | 0.762 | 0 | 0 | 0 | 0 | 0 |
| WCST_P | 0 | -0.070 | 0 | 0 | -0.040 | 0 | 0 | 0 | 0 | 0 | 0.443 | 0 | 0 |
| WCST_C | 0 | 0 | 0 | 0 | -0.089 | 0 | 0 | 0 | 0 | 0.443 | 0 | 0 | 0 |
| IQ_S | 0 | 0 | 0 | -0.128 | -0.067 | 0.169 | 0 | 0 | 0 | 0 | 0 | 0 | 0.472 |
| IQ_B | 0 | 0 | 0 | 0 | -0.025 | 0 | 0 | 0 | 0 | 0 | 0 | 0.472 | 0 |

**Table S16.** Overview of the edges between specific cognitive functions domains and clinical-glycemic characteristics in the network for childhood-onset adult patients (N =181. The total strength in the table is the absolute value.

|  | | | | | | |
| --- | --- | --- | --- | --- | --- | --- |
|  | Total edges | Positive edges | Negative edges | Total strength | Positive strength | Negative strength |
| All edges | 16 | 8 | 8 | 3.916 | 3.047 | -0.869 |
| Between cognitive functions nodes | 5 | 5 | 0 | 1.696 | 1.696 | 0 |
| Between clinical-glycemic nodes | 4 | 2 | 2 | 0.900 | 0.450 | -0.450 |
| Interaction between cognitive functions and clinical-glycemic characteristics | 7 | 1 | 6 | 0.513 | 0.093 | -0.420 |
|  |  |  |  |  |  |  |
